# Charge-based interactions through peptide position 4 drive diversity of antigen presentation by human leukocyte antigen class I molecules

**DOI:** 10.1093/pnasnexus/pgac124

**Published:** 2022-07-27

**Authors:** Kyle R Jackson, Dinler A Antunes, Amjad H Talukder, Ariana R Maleki, Kano Amagai, Avery Salmon, Arjun S Katailiha, Yulun Chiu, Romanos Fasoulis, Maurício Menegatti Rigo, Jayvee R Abella, Brenda D Melendez, Fenge Li, Yimo Sun, Heather M Sonnemann, Vladislav Belousov, Felix Frenkel, Sune Justesen, Aman Makaju, Yang Liu, David Horn, Daniel Lopez-Ferrer, Andreas F Huhmer, Patrick Hwu, Jason Roszik, David Hawke, Lydia E Kavraki, Gregory Lizée

**Affiliations:** University of Texas MD Anderson Cancer Center, UTHealth Graduate School of Biomedical Sciences, Houston, TX, USA; Department of Melanoma, UT MD Anderson Cancer Center, Houston, TX, USA; Department of Biology and Biochemistry, University of Houston, Houston, TX, USA; Department of Computer Science, Rice University, Houston, TX, USA; Department of Melanoma, UT MD Anderson Cancer Center, Houston, TX, USA; Department of Melanoma, UT MD Anderson Cancer Center, Houston, TX, USA; Department of Melanoma, UT MD Anderson Cancer Center, Houston, TX, USA; University of Texas MD Anderson Cancer Center, UTHealth Graduate School of Biomedical Sciences, Houston, TX, USA; Department of Immunology, UT MD Anderson Cancer Center, Houston, TX, USA; Department of Melanoma, UT MD Anderson Cancer Center, Houston, TX, USA; Department of Melanoma, UT MD Anderson Cancer Center, Houston, TX, USA; Department of Computer Science, Rice University, Houston, TX, USA; Department of Computer Science, Rice University, Houston, TX, USA; Department of Computer Science, Rice University, Houston, TX, USA; University of Texas MD Anderson Cancer Center, UTHealth Graduate School of Biomedical Sciences, Houston, TX, USA; Department of Melanoma, UT MD Anderson Cancer Center, Houston, TX, USA; Department of Melanoma, UT MD Anderson Cancer Center, Houston, TX, USA; University of Texas MD Anderson Cancer Center, UTHealth Graduate School of Biomedical Sciences, Houston, TX, USA; Department of Melanoma, UT MD Anderson Cancer Center, Houston, TX, USA; University of Texas MD Anderson Cancer Center, UTHealth Graduate School of Biomedical Sciences, Houston, TX, USA; Department of Melanoma, UT MD Anderson Cancer Center, Houston, TX, USA; BostonGene Corporation, Waltham, MA, USA; BostonGene Corporation, Waltham, MA, USA; Immunitrack Aps, Copenhagen, Denmark; ThermoFisher Scientific, San Jose, CA, USA; ThermoFisher Scientific, San Jose, CA, USA; ThermoFisher Scientific, San Jose, CA, USA; ThermoFisher Scientific, San Jose, CA, USA; ThermoFisher Scientific, San Jose, CA, USA; Department of Melanoma, UT MD Anderson Cancer Center, Houston, TX, USA; Department of Melanoma, UT MD Anderson Cancer Center, Houston, TX, USA; Department of Systems Biology, UT MD Anderson Cancer Center, Houston, TX, USA; Department of Computer Science, Rice University, Houston, TX, USA; Department of Melanoma, UT MD Anderson Cancer Center, Houston, TX, USA; Department of Immunology, UT MD Anderson Cancer Center, Houston, TX, USA

**Keywords:** peptide antigen presentation, human leukocyte antigen (HLA), mass spectrometry, computational modeling, molecular dynamics

## Abstract

Human leukocyte antigen class I (HLA-I) molecules bind and present peptides at the cell surface to facilitate the induction of appropriate CD8+ T cell-mediated immune responses to pathogen- and self-derived proteins. The HLA-I peptide-binding cleft contains dominant anchor sites in the B and F pockets that interact primarily with amino acids at peptide position 2 and the C-terminus, respectively. Nonpocket peptide–HLA interactions also contribute to peptide binding and stability, but these secondary interactions are thought to be unique to individual HLA allotypes or to specific peptide antigens. Here, we show that two positively charged residues located near the top of peptide-binding cleft facilitate interactions with negatively charged residues at position 4 of presented peptides, which occur at elevated frequencies across most HLA-I allotypes. Loss of these interactions was shown to impair HLA-I/peptide binding and complex stability, as demonstrated by both in vitro and in silico experiments. Furthermore, mutation of these Arginine-65 (R65) and/or Lysine-66 (K66) residues in HLA-A*02:01 and A*24:02 significantly reduced HLA-I cell surface expression while also reducing the diversity of the presented peptide repertoire by up to 5-fold. The impact of the R65 mutation demonstrates that nonpocket HLA-I/peptide interactions can constitute anchor motifs that exert an unexpectedly broad influence on HLA-I-mediated antigen presentation. These findings provide fundamental insights into peptide antigen binding that could broadly inform epitope discovery in the context of viral vaccine development and cancer immunotherapy.

Significance Statement Intracellular peptides complexed to human leukocyte antigen Class I (HLA-I) molecules are critical for directing appropriate cellular immunity mediated by T lymphocytes. This study describes additional conserved features of HLA-I molecules that drive binding, stability, and diversity of the presented peptide repertoire. Nonpocket interactions between specific prevalent HLA-I residues and peptide positions 1 and 4 make unexpectedly strong contributions to peptide stabilization and antigen presentation. These interactions are shared across the HLA-I system and other species, and may constitute a subdominant anchor motif. Better understanding of the role of subdominant interactions and the individual contributions of nonpocket residues toward peptide binding, as described here, can be leveraged to create more accurate and generalizable methods for identifying targets of T-cell mediated immune responses.

**List of abbreviations**
HLA-I: Human leukocyte antigen class I
MHC-I: Major histocompatibility complex class I
TCR: T-cell receptor
CTL: Cytotoxic T lymphocytes
MS: Mass spectrometry
p4: Peptide position 4 amino acid
pΩ: Peptide C-terminal amino acid
MD: Molecular dynamics
β2m: Beta-2 microglobulin

## Background

Major histocompatibility complex class I (MHC-I) molecules are expressed at the surface of nearly all nucleated vertebrate cells, where they each present a diverse array of peptides of 8 to 13 amino acids in length sampled from recently synthesized cellular proteins (1). This process is crucial for immune surveillance by CD8+ T lymphocytes, which can recognize MHC-I/peptide complexes of target cells through specific molecular interactions with T-cell receptors (TCRs) (2, 3). Peptide antigen presentation by MHC-I can cause activation of cytotoxic T-cell lymphocytes (CTL) against pathogen-derived peptides leading to pathogen clearance, or to induction of T-cell tolerance. MHC-I genes, known collectively as human leukocyte antigens Class I (HLA-I) in humans, are highly polymorphic, with most of the variability occurring within the peptide-binding region and resulting in widely disparate peptide-binding preferences amongst HLA-I allotypes (4–7). The enormous breadth of HLA-I presented peptides complements the immense TCR diversity generated by genetic recombination events in CD8+ T lymphocytes (8–11). As central targets of therapeutic CTL responses against disease, defining the key molecular features that determine peptide ligand interactions with the many thousands of HLA-I allotypes present within the human population is currently of great interest to immunologists and clinicians alike (12–14).

Mass spectrometric (MS) analysis of HLA-I peptide ligands eluted directly from human cell lines and tissue samples has enabled the identification of peptide-binding motifs for >100 HLA-I molecules, which has facilitated the development of peptide binding prediction algorithms with very good accuracy and demonstrated clinical utility (15–21). HLA-I peptide-binding motifs share a strong conservation of particular amino acid residues at the peptide N- (residues p1 to p3) and C-termini, which constitute dominate “end-anchors” essential for peptide binding that interact with pockets at the bottom of the peptide-binding cleft (22–24). With a few exceptions (e.g. HLA-B*0801), residues in the middle of the bound peptide (residues p4 to p-1) are generally located near the top of the binding cleft or bulging outward and away from the cleft, and are primarily recognized by cognate TCRs (25–29). A number of studies have demonstrated that allotype-specific interactions also occur between middle peptide residues and HLA-I residues in secondary pockets or along the inner sides of the *α*-helices that form the binding cleft (30–35). However, most of these studies were limited in scope and none directly analyzed the broader contributions of these secondary interactions in the context of natural antigen processing and presentation by cells.

To identify such secondary interactions, we conducted a comprehensive survey of HLA-I peptide ligands and observed significantly elevated frequencies of negatively charged amino acids aspartic acid (D) and glutamic acid (E) at peptide position 4 (p4), with the highest prevalence found in the HLA-A2 family. Crystal structures of A*02:01, the most prevalent HLA-A2 allotype, revealed that D/E4 was located near the top of the binding cleft adjacent to two positively charged HLA-I residues, indicating a potential for ionic interactions (36–38). This agreed with previous studies showing that A*02:01 bound peptides containing phosphate moieties at p4 that also interacted with these positively charged residues (31). To assess the contribution of these putative interactions for overall antigen presentation, we mutated HLA-A*02:01 and another highly prevalent HLA-A allotype, A*24:02, to include or exclude these positively charged HLA-I residues. Peptides eluted from wild-type (WT) and mutated HLA-I molecules were analyzed for differences, while peptide binding and stability assays were performed on both WT A*02:01 and A*24:02 molecules. Computational analyses, including molecular dynamics (MD) simulations, were also performed to provide mechanistic insights. Collectively, the results demonstrate that HLA-I residues Arginine-65 (R65) and Lysine-66 (K66) facilitate stabilization of charge-based interactions with D/E4 residues of bound peptide antigens and are crucial for optimal antigen presentation and complete diversity of the presented peptide repertoire. Our study sheds light on the distinct contributions made by R65 and K66 and reveals new insights regarding the nature of N-terminal (p1-3) and C-terminal (pΩ) peptide binding that have broad relevance for antigen presentation throughout the classical MHC-I system.

## Results

### HLA class I molecules demonstrate a preference for acidic residues at peptide position 4

**Fig. 1.**
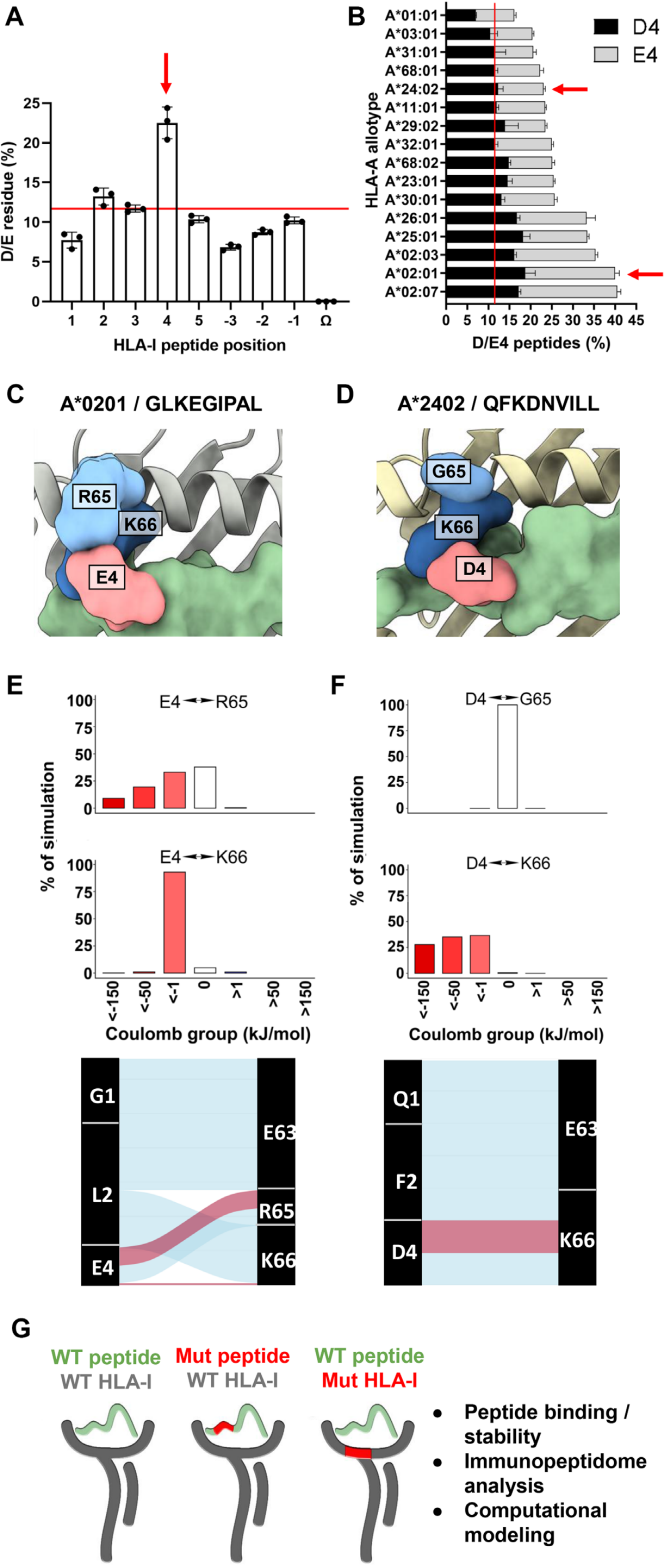
HLA class I prefers acidic residues at peptide position 4 found in close proximity to basic residues in positions 65/66. (A) Frequency of aspartic acid (D) and glutamic acid (E) residues at all HLA-I peptide positions (N-terminus p1 to p5 and C-terminus pΩ to p−3) from 108 HLA-I allotypes, where each dot represents one of three public peptide databases and the red line indicates the background frequency of D/E residues found in vertebrate proteins (∼12%). (B) Frequency of D/E4 peptides eluted from 16 prevalent HLA-A allotypes from three different peptide databases. Red arrows highlight HLA-A allotypes A*02:01 and A*24:02 that were the main focus of this study. (C)/(D) Top view from crystal structures of HLA-I/peptide complexes A*02:01/GLKEGIPAL (C) and A*24:02/QFKDNVILL (D), where the peptide is green. (E)/(F) Top panels: coulombic interactions between peptide p4 and HLA-I p65 or p66 from MD simulations of A*02:01/GLKEGIPAL (E) and A*24:02/QFKDNVILL (F) complexes, where negative values are red and positive values are blue. Bottom panels: alluvial plots showing selected interactions formed during simulations of A*02:01/GLKEGIPAL (E) and A*24:02/QFKDNVILL (F). Blue lines indicate hydrogen bonds, red lines indicate salt bridges, and width indicates proportional prevalence. (G) Schematic showing the overall strategy and the experimental approaches utilized in this study.

To identify potential secondary interactions mediating HLA-I-mediated antigen presentation, we performed a comprehensive search of MS-eluted peptides from 108 HLA-I allotypes to assess whether specific amino acid residues were preferred in the middle positions of peptide ligands (i.e. positions p4 to p-1). We found a striking preference for the negatively charged residues aspartic acid (D) and glutamic acid (E) at peptide position 4 (p4) for most HLA-I allotypes (Fig. 1A). Further analysis showed that the frequency of D/E at p4 was significantly higher (mean: 25%, range: 18% to 42%) than the frequency of D/E residues found in vertebrate proteins (11.7%), a trend that was observed across all databases (Figure S1A, Supplementary Material). Elevated D/E4 frequencies were also observed in MHC-I-bound peptides from four other animal species analyzed, suggesting that it may be a conserved feature of MHC-I peptide ligands (Figure S1B, Supplementary Material). Peptides eluted from HLA-A, B, and C allotypes showed significantly elevated frequencies of D/E4 residues, with the following hierarchy: HLA-A > HLA-C > HLA-B (Figure S1C, Supplementary Material). Closer examination of p4 residues revealed no clear preference for any other amino acids with the exception of proline, which was the most frequent p4 residue amongst HLA-B and -C ligands (Figures S1D–S1F, Supplementary Material). Different HLA-I allotypes demonstrated a range of preferences for D/E4 residues, with the HLA-A2 family of allotypes showing the highest frequencies overall (Fig. 1B; Figures S2–S5, Supplementary Material). Taken together, these data show a significant bias amongst eluted MHC-I peptide ligands that favors the presence of negatively charged amino acid residues D or E at position p4.

Based on these observations, we hypothesized that the high prevalence of D/E at p4 is driven by specific interactions with residues in the HLA peptide-binding cleft. The superposition of multiple crystal structures of A*02:01-restricted D/E4-containing peptides revealed that D/E4 interacted primarily with the basic arginine residue R65 of A*02:01, though some interactions also occurred with the basic lysine residue K66 (Figure S6A, Supplementary Material). By contrast, examination of crystal structures of A*02:01 complexed with peptides containing proline at position 4 (P4) showed no proline-mediated interactions with R65, K66, or any other HLA-I residues (Figure S6B, Supplementary Material). Notably, recent Markov state modeling studies of peptides containing P4 indicated that proline increased peptide rigidity, and thus HLA-I/peptide stability without interacting with adjacent residues (39). Therefore, we focused our studies on investigating the potential charged interactions between peptide D/E4 residues and basic HLA-I residues at positions 65 and 66 in A*02:01 and A*24:02, two of the most prevalent HLA-A molecules worldwide (Figures S6C, S6D and Tables S1 and S2, Supplementary Material).

To gain further insights into the roles of R65 and K66 in peptide binding, we performed MD simulations on crystal structures of A*02:01/GLKEGIPAL and A*24:02/QFKDNVILL complexes (Videos S1 and S2, Supplementary Material). For both complexes, starting crystal structures (Fig. 1C and D) and MD simulations (Figures S6E and S6F, Supplementary Material) showed that peptide position 4 is in much closer proximity to R65 and K66 on the *α*1 helix residues than to residues on the opposite *α*2 helix. In A*02:01/GLKEGIPAL, R65 showed extensive interactions solely with E4 at the top of the peptide binding cleft while K66, located on the inner side of the cleft, interacted in a network with peptide residues L2 and E4 in addition to HLA residues E63 and H70 (Figure S6G, Supplementary Material). In A*24:02/QFKDNVILL, K66 also formed network of interactions with peptide residues F2 and D4 and HLA residues E62 and E63, while no significant peptide interactions were observed to occur with G65 (Figure S6H, Supplementary Material). Calculation of coulombic interactions and hydrogen bonds between E4 of GLKEGIPAL and R65/K66 of A*02:01 revealed salt bridges with both basic HLA-I residues, though R65 exhibited more stable salt bridges with E4 (Fig. 1E; Figure S7A, Supplementary Material). By contrast, MD simulations of A*02:01 bound to the peptides VLHDDLLEA and FLKEPGHGV showed that K66 formed more stable salt bridges with D/E4 than R65, indicating that preferences for interactions with R65 or K66 are peptide-specific (Figures S7B and S7C, Supplementary Material). MD analysis of A*24:02 bound to QFKDNVILL showed strong charged interactions and formation of salt bridges between peptide residue D4 and K66 (Fig. 1F; Figure S7D, Supplementary Material). To exclude the potential bias of only using peptides with D/E4 as reference for our MD simulations, we conducted a control experiment based on four crystal structures of peptides having other prevalent residues at p4 (Figure S8, Supplementary Material). For each of these systems, we replaced the WT p4 residue (P4, S4, or F4) with A4, D4, or E4. Our results across both A*02:01- and A*24:02-restricted complexes demonstrated a lack of direct HLA interactions with P4 and S4, and the consistent addition of salt-bridges and hydrogen bonds with D/E4. Interestingly, F4 was also observed to interact with K66 through Pi–Cation interactions, demonstrating that other amino acids in this position can also contribute to HLA-I binding. To empirically test these proposed molecular interactions, we created a series of HLA-A*02:01 and A*24:02 mutant constructs that altered positions 65 and/or 66. Comparative MS-based immunopeptidome analysis was then performed on eluted peptides. To validate potential secondary interactions and gain further molecular insights, synthetic peptide binding and stability assays were performed using WT A*02:01 and A*24:02 molecules, and computational simulations were done on both WT and mutated A*02:01 and A*24:02 molecules (Fig. 1G).

### Basic residue substitutions in HLA-A*02:01 reduce surface expression, peptide repertoire diversity, and presentation of D/E4 ligands

**Fig. 2.**
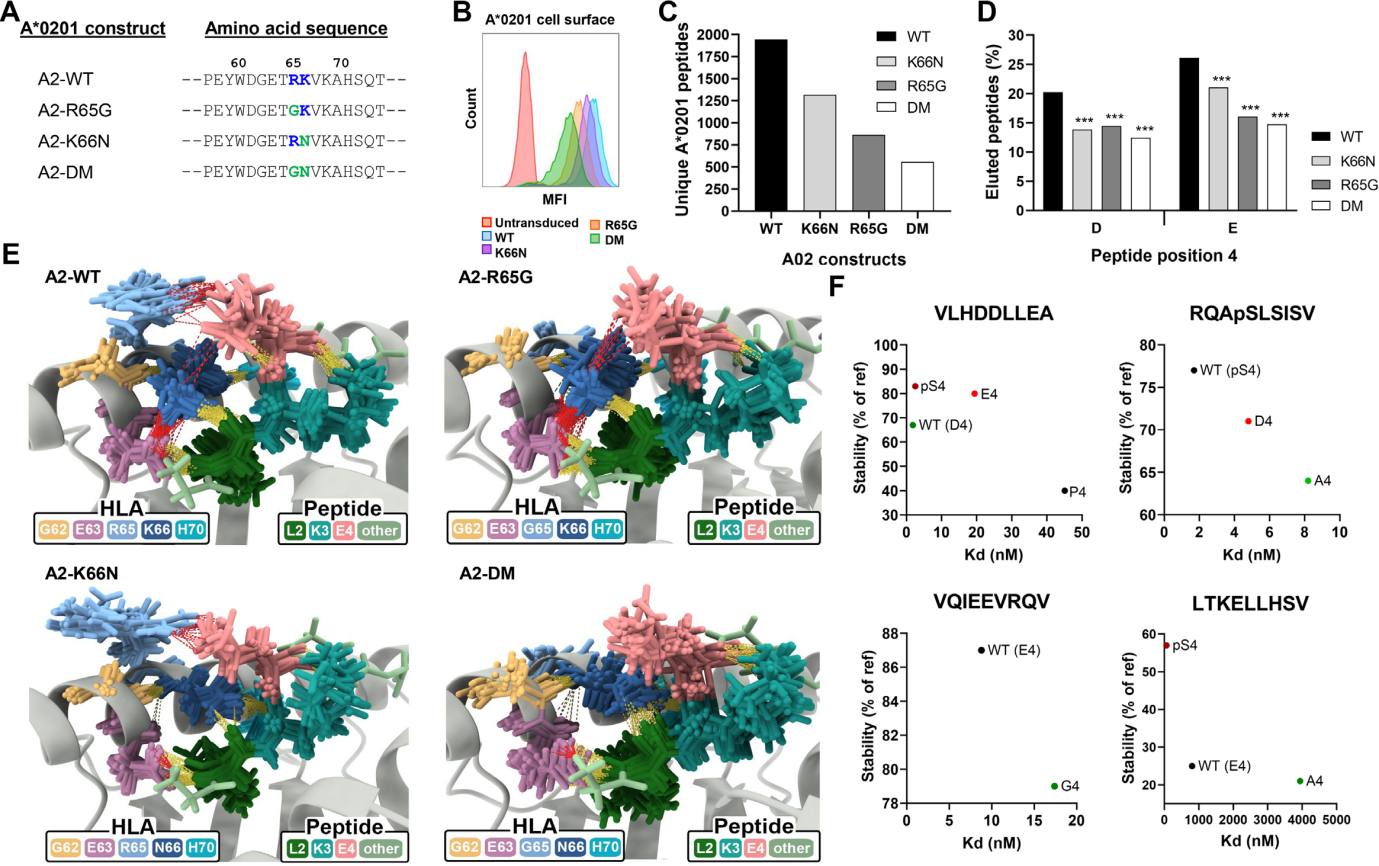
Basic p65/66 residue substitutions to HLA-A*02:01 reduce surface expression, peptide repertoire diversity, and presentation of D/E4 ligands. (A) Amino acid positions 57 to 73 of WT HLA-A*02:01 and the three p65/66-mutated A*02:01 molecules, where positively charged residues are in blue and substituted residues are green at p65 and p66. (B) Cell surface expression of A*02:01 WT or mutant molecules in transduced H1975 lung cancer cells, as determined by flow cytometry. (C) Total number of unique peptides identified by mass spectrometry of A*02:01 molecules eluted from WT or mutant transduced H1975 cells. (D) Proportion of peptides containing D or E in peptide position 4 in A*02:01 WT or mutant molecules. (E) Ensemble of representative conformations extracted from multiple MD simulations of GLKEGIPAL bound to A2-WT, A2-R65G, A2-K66N, and A2-DM, where yellow dashed lines indicate hydrogen bonds and red dashed lines indicate salt bridges. (F) Peptide binding and stability assays performed on four peptides with various p4 substitutions in WT A*02:01 molecules. Stability values were calculated compared to the reference peptide LLFGYPVYV. *** indicates *P* ≤ 0.001 using a two proportion Z-test comparing amino acid frequencies of peptides eluted from mutant molecules to frequencies of peptides eluted from WT HLA-A*02:01.

To investigate the role of R65 and K66 in antigen presentation, three different HLA-A*02:01 mutant alleles were created that altered either one or both residues (Fig. 2A; Figure S9A, Supplementary Material). Arginine in position 65 was substituted with glycine to create A*02:01-R65G (A2-R65G), resulting in the G65/K66 pairing that is found naturally in both HLA-A*02:70 and A*24:02. The second mutant changed position 66 from lysine to asparagine to create A*02:01-K66N (A2-K66N) resulting in the HLA-A consensus configuration R65/N66, which is also found naturally in the HLA-A*02:20 allele. The third mutant combined both p65/66 mutations to create the A*02:01-R65G/K66N double mutant (A2-DM) featuring the G65/N66 combination, which does not occur naturally in any known HLA-A2 family allele (40–42).

Recombinant lentiviruses expressing WT HLA-A*02:01 (A2-WT) or the three mutant A*02:01 alleles with green fluorescent protein (GFP) tagged onto the C-terminus were used to transduce the lung cancer cell line H1975. Transduced cells demonstrating comparable levels of total GFP expression (Figure S9B, Supplementary Material) showed strikingly different levels of A*02:01 cell surface expression (Fig. 2B). While A2-WT exhibited the highest surface expression, the three mutated A*02:01 molecules consistently demonstrated reduced expression with the following hierarchy: A2-WT > A2-K66N > A2-R65G > A2-DM, with A2-DM showing ∼ 80% reduction compared to A2-WT. To further explore these deficiencies in cell surface expression, we performed mass spectrometry (MS)-based immunopeptidome analysis to compare the quality and quantity of peptides bound to the different A*02:01 variants. As shown in Fig. 2(C), all three A2 mutants demonstrated decreased total numbers of unique A*02:01-restricted peptides compared to A2-WT. This loss of peptide diversity correlated well with loss of surface expression, with A2-DM showing a > 70% reduction in the numbers of unique eluted peptides compared with A2-WT (Fig. 2C). Thus, loss of either one of the basic residues at p65/66 caused a significant reduction in HLA-A*02:01 cell surface presentation and peptide repertoire diversity, with substitutions to both basic residues having the strongest impact.

Based on the A*02:01/peptide crystal structure-based evidence, we hypothesized that the loss of the interaction between peptide D/E4 residues and R65/K66 in A*02:01 may have been responsible for the loss of peptide repertoire and cell surface expression observed for the A*02:01 mutants. Analysis of the frequency of D/E4 residues in peptides eluted from the A*02:01 mutants indeed showed a significant loss of presented D4 and E4 peptides (*P* < 0.001; Fig. 2D). D4 peptide frequencies were reduced by ∼ 35% in all three A*02:01 mutants, while E4 peptides were reduced by ∼ 50% for both A2-R65G and A2-DM and ∼ 25% for A2-K66N. Corresponding increases were observed in other amino acids at position 4 including proline, alanine, serine, and glycine (Figure S9C, Supplementary Material).

To further analyze interactions between peptide D/E4 residues and R65/K66, MD simulations were run of the peptide GLKEGIPAL bound to A2-WT or each of the three A2 mutants (Videos S1, S3, S4, and S5, Supplementary Material). Strong negative coulombic interactions between E4 and R65 in A2-WT were disrupted in the A2-R65G and A2-DM mutants but not in the A2-K66N mutant (Figures S10A–S10D, Supplementary Material). Salt bridge interactions increased between E4 and K66 in A2-R65G compared to A2-WT, indicating that K66 can compensate somewhat for the loss of R65 (Figures S10A and S10B, Supplementary Material). Superposition of the most prevalent structures from each MD simulation revealed salt bridges between E4/K66 in A2-R65G and E4/R65 in A2-K66N, but both of these interactions were lost in A2-DM (Fig. 2E). R65 interacted primarily with peptide position 4 residues (Fig. 2E; Figure S6A, Supplementary Material) while K66 was involved in multiple additional interactions, primarily hydrogen bonding with the backbone of L2 and forming salt bridges with E63, both of which were reduced or abrogated with the K66N substitution (Fig. 2E). In fact, most interactions between E4 and other residues were lost in the most prevalent MD structures from A2-DM (Fig. 2E). However, in a small number of conformations from A2-K66N and A2-DM, salt bridges were observed between N66 and E4, though they were much less stable than those between K66 and E4 in A2-WT (Figures S10C and S10D, Supplementary Material). Further analysis revealed that the amide group of N66 can either attract or repel the side chain of E4 leading to these weak interactions (Figures S10C and S10D, Supplementary Material). Examination of all conformations from all MD simulations showed decreased hydrogen bond and salt bridge-mediated interactions with peptide position 4 as R65 and/or K66 were lost (Figure S10E, Supplementary Material).

To directly evaluate the contribution of peptide p4 residues to antigen presentation, we performed peptide binding and stability assays on WT A*02:01 molecules using a panel of synthetic p4 residue-substituted peptides. Using four different peptide backbones, these assays showed that highly negatively charged p4 phospho-serine residues imparted the highest levels of binding and stability, followed closely by D4 and E4 (Fig. 2F). By contrast, p4 substitutions with common A*02:01 residues such as alanine or glycine resulted in decreased peptide binding and stability (Fig. 2F). MD simulations of A*02:01/GLKEGIPAL showed that salt bridges occurred primarily between E4 and R65; substitution to D4 maintained this interaction with R65 while further promoting salt bridge formation with K66 (Figure S10F and Video S6, Supplementary Material). As expected, alteration of position 4 to alanine led to loss of all salt bridges, consistent with the loss of binding and stability observed with p4 residue-substituted peptides (Figure S10F and Video S7, Supplementary Material). Collectively, these results demonstrate that charged interactions between peptide D/E4 residues and R65/K66 make a substantial contribution to peptide binding, stability, and antigen presentation in HLA-A*02:01.

### Basic residue substitutions in HLA-A*02:01 result in peptide-binding motif changes impacting position 1 and primary anchor residues

**Fig. 3.**
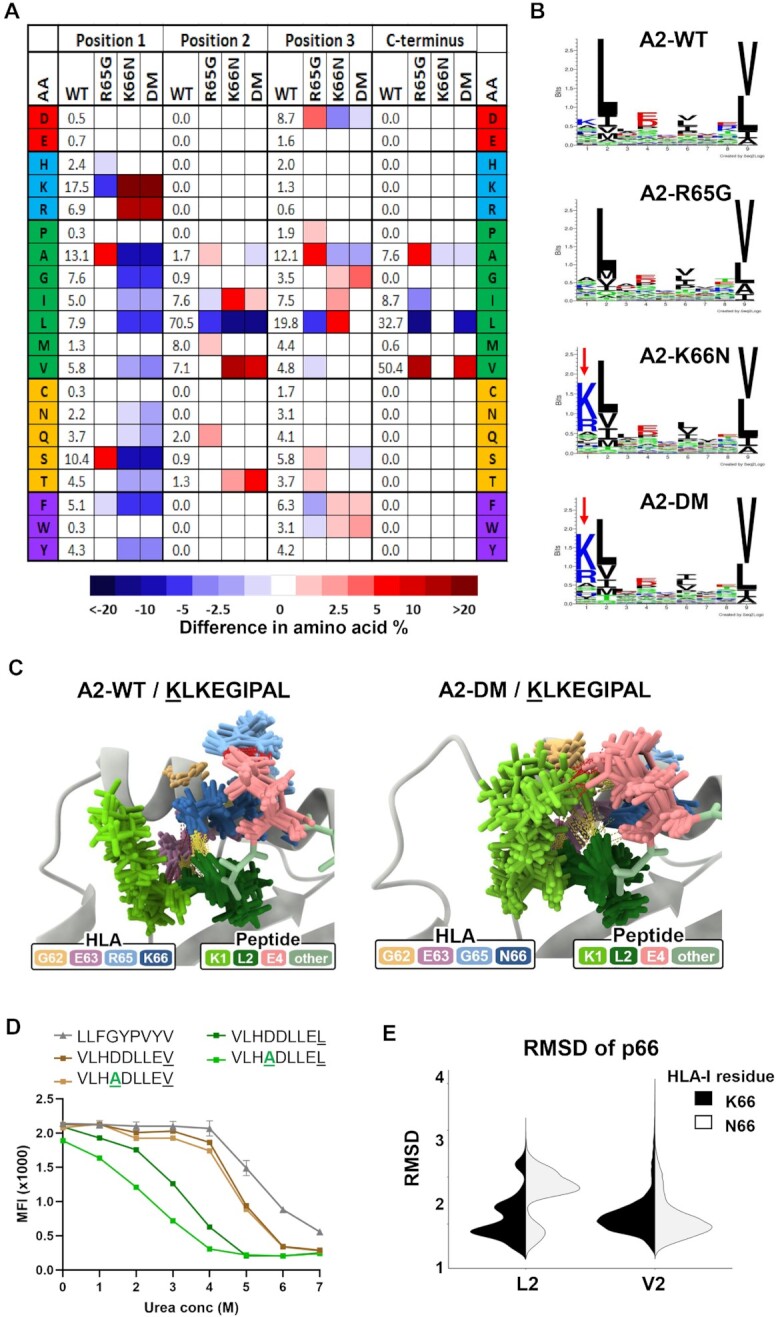
Changes to basic p65/66 residues of A*02:01 impacts peptide position 1 and primary anchor residues of eluted peptide ligands. (A) Heat map depicting the changes in frequencies for all 20 amino acids at positions 1 to 3 and the C-terminus (pΩ) in A2 mutants compared to WT A*02:01. (B) Peptide-binding motifs derived from peptides eluted from A2-WT, A2-R65G, A2-K66N, or A2-DM molecules generated by Seq2Logo. Red arrows indicate the increased frequency of positively charged amino acids in position 1. (C) Ensemble of representative conformations extracted from multiple MD simulations of the p1-substituted KLKEGIPAL peptide bound to A2-WT or A2-DM, where yellow dashed lines indicate hydrogen bonds and red dashed lines indicate salt bridges. (D) Peptide stability assays comparing p4 and/or pΩ residue-substituted peptides VLHDDLLEV, VLHADLLEV, VLHDDLLEL, and VLHADLLEL bound to WT HLA-A*02:01. (E) Root Mean Square Deviation (RMSD) of the HLA-I residue K66 or N66 of all conformations extracted from MD simulations of GLKEGIPAL and GVKEGIPAL bound to A2-WT and A2-K66N and crystallographic conformations of p66 in A*02:01/GLKEGIPAL (PDB code 5ENW).

In addition to inducing changes in peptide repertoire diversity and frequency of eluted D/E4 peptides, substitutions to R65 and/or K66 induced other significant immunopeptidome changes (Fig. 3A; Figure S11A, Supplementary Material). The most striking change occurred in position 1 of peptides (p1) eluted from both the A2-K66N and A2-DM mutants: while only 25% of A2-WT peptides contained positively charged residues lysine (K) or arginine (R) at p1, peptides eluted from A2-K66N and A2-DM demonstrated a K/R1 frequency of greater than 70% (Fig. 3B; Figure S11B, Supplementary Material). To explore the reasons for these p1 frequency shifts, MD simulations of A*02:01/GLKEGIPAL complexes were repeated in which K1 was substituted for G1 (Video S8, Supplementary Material). Interestingly, this alteration resulted in the formation of more prevalent salt bridges between E4-R65 and E4-K66, which should contribute to improved stability (Figure S12A, Supplementary Material). Consistent with this, we also observed increased stability by substituting V1 with K1 in VLHDDLLEA bound to A*02:01 (Figure S12B, Supplementary Material).

To better understand the dramatic increases in K/R1 peptide frequencies observed in the A2-K66N and A2-DM mutants, we ran MD simulations of the p1-substituted KLKEGIPAL peptide bound to all three A*02:01 variants (Videos S9–S11, Supplementary Material). Since interactions between K1 and A*02:01 residues (Figure S12C, Supplementary Material) did not explain the strong preference for K/R1 peptides, we examined potential interactions between K1 and other amino acids within the KLKEGIPAL peptide. Interestingly, in A2-K66N and A2-DM a salt bridge interaction between K1 and E4 was observed in 25% of all sampled MD conformations, compared to only ∼ 2% of conformations for A2-WT (Fig. 3C; Figure S12D, Supplementary Material). This increased prevalence of an intrapeptide salt bridge between K1 and E4 could explain the preference for K/R1 in these mutants. This notion was supported by additional analyses showing that K/R1-containing peptides eluted from all A*02:01 variants showed a significant degree of co-occurrence with D/E4 residues, and vice-versa (Figure S12E, Supplementary Material). Further analysis of peptides containing K/R1 and D/E4 from multiple HLA-A, -B, and -C allotypes revealed a significant correlation between amino acids in these positions, indicating that these amino acids co-occur throughout the HLA-I system (Figure S12F and Database S1, Supplementary Material).

Peptides eluted from A*02:01 mutants also showed alterations in the frequency of primary anchor residues at p2 and the C-terminus (pΩ). The impact of these mutations on pΩ was particularly surprising since p65/66 residues are relatively distant from the F pocket which harbors pΩ residues. Nonetheless, peptides eluted from both A2-R65G and A2-DM showed a significant preference for valine residues at pΩ with a corresponding decreased preference for leucine (LΩ) compared to A2-WT. (Fig. 3A; Figure S13A, Supplementary Material). VΩ is more prevalent than LΩ and MD simulations with GLKEGIPAL and GLKEGIPAV showed more extensive hydrogen bonds and shorter mean distance to the floor for VΩ than LΩ in A2-WT and all mutated variants (Figures S13B and S13C, Supplementary Material). We therefore reasoned that charged interactions between p4 and p65/66 might be more important for stabilizing LΩ containing peptides than VΩ peptides. To test this, A*02:01 peptide stability assays were performed on D4A-substituted variants of VLHDDLLEV and VLHDDLLEL. As shown in Fig. 3(D), VΩ provided more stability than LΩ, and altering D4 to A4 destabilized the LΩ peptide while having a negligible impact on stability of the VΩ peptide. This result supports the idea that charged interactions of A*02:01 with D/E4 residues are more important for stabilizing peptides with weaker pΩ anchors.

In peptides eluted from the A2-K66N and A2-DM mutants, we also observed alterations to amino acid frequencies in peptide position 2 (p2). These changes were not surprising since several A*02:01/peptide crystal structures have shown K66 consistently forming hydrogen bonds with the backbone of p2 residues (Figures S6A, S6G, S7A–S7C, Supplementary Material). Peptides eluted from both A2-K66N and A2-DM demonstrated a 20% decrease in the frequency of leucine (L2), the most dominant p2 anchor, with a corresponding increase in valine (V2) residues compared to A2-WT peptides (Fig. 3A; Figure S13D, Supplementary Material). Comparative MD simulations of GLKEGIPAL and GVKEGIPAL peptides showed that while both formed similar hydrogen bonds, V2 was closer to the beta-floor sheet than L2, consistent with tighter binding in the B pocket and improved peptide stability (Figures S13E and S13F, Supplementary Material) (43, 44). Furthermore, comparison of the relative movements of p66 residues indicated that N66 was more mobile with L2 but more stable with V2 present; by contrast, K66 made stable interactions with either L2 or V2 (Fig. 3E), providing a potential explanation for the V2 preference of the A2-K66N and A2-DM molecules.

### Basic residue substitutions in HLA-A*24:02 impact surface expression, peptide repertoire diversity, and presentation of D/E4 ligands

**Fig. 4.**
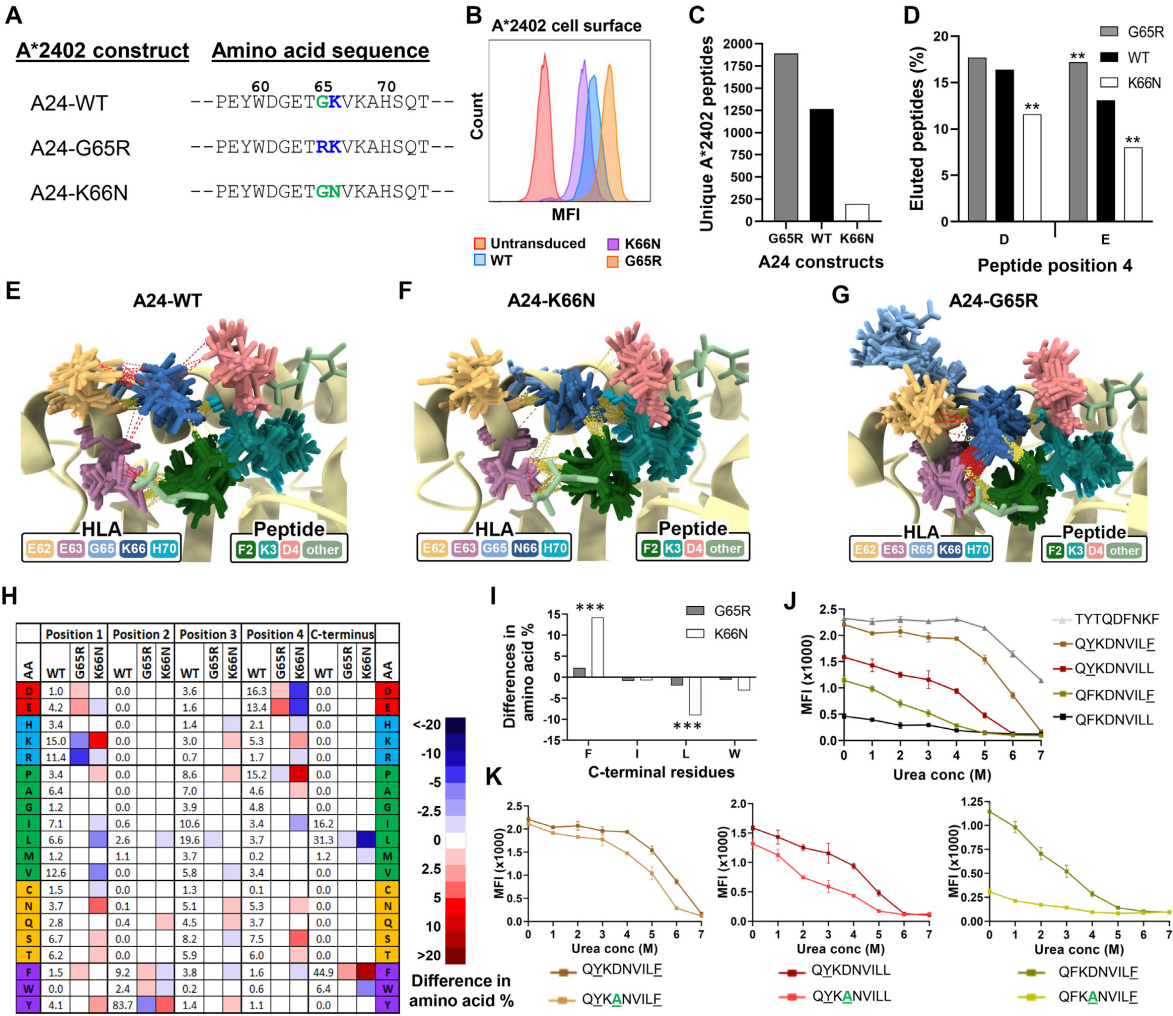
Basic residues in p65/66 of HLA-A*24:02 determine cell surface expression levels, peptide repertoire diversity, and binding stability. (A) Amino acid positions 57 to 73 of WT HLA-A*24:02 and both of the mutated A*24:02 molecules, where positively charged residues are in blue and substituted residues are in green at p65 and p66. (B) Cell surface expression of WT A*24:02 or mutant molecules in transduced H1975 lung cancer cells, as determined by flow cytometry. (C) Total number of unique peptide sequences identified by MS analysis of peptide ligands eluted from WT A*24:02 or mutant molecules immunoprecipitated from transduced H1975 cells. (D) Proportion of peptides containing D or E in peptide position 4 in A*24:02 WT or mutant molecules. (E)–(G) Ensemble of representative conformations extracted from multiple MD simulations of QFKDNVILL bound to A24-WT, A24-K66N, and A2-DM, where yellow dashed lines indicate hydrogen bonds and red dashed lines indicate salt bridges. (H) Heat map depicting the changes in amino acid frequencies at peptide positions 1 to 4 and pΩ in A24 mutants compared to WT A*24:02. (I) Differences in the amino acid frequencies of C-terminal (pΩ) anchor residues in peptides eluted from A24-G65R or A24-K66N mutants compared to A24-WT. (J) Results of peptide stability assays comparing the parent QFKDNVILL peptide with the p2 and/or pΩ residue-substituted peptides QYKDNVILL, QFKDNVILF, and QYKDNVILF. For these assays, TYTQDFNKF was used as a positive control and reference peptide. (K) Peptide stability assays of the QYKDNVILF, QYKDNVILL, and QFKDNVILF peptides compared with their D4A-substituted counterparts, where TYTQDFNKF was used as a reference peptide. ** indicates *P* ≤ 0.01 and *** indicates *P* ≤ 0.001 using a two proportion Z-test comparing amino acid frequencies of peptides eluted from mutant molecules to frequencies eluted from WT A*24:02.

We next determined the impact of basic residue alterations at p65/66 for HLA-A*24:02. Since A*24:02 naturally contains only one basic residue at K66, two different A*24:02 mutant alleles were created: the lysine in p66 was substituted with asparagine to create A*24:02-K66N (A24-K66N), resulting in the G65/N66 pairing that was a feature of the A2-DM mutant described above, and that was also identical in sequence to the known allele HLA-A*24:145. The second A*24:02 mutant changed the glycine at p65 to arginine to create A*24:02-G65R (A24-G65R), recapitulating the R65/K66 pairing found in WT A*02:01, and identical in sequence to HLA-A*24:29 (Fig. 4A; Figure S14A, Supplementary Material) (4).

Recombinant lentiviruses expressing WT HLA-A*24:02 (A24-WT) or the two A*24:02 mutants were used to transduce the lung cancer cell line H1975. Similar to HLA-A*02:01, the A*24:02 mutants consistently demonstrated distinct cell surface expression levels with the following hierarchy: A24-G65R > A24-WT > A24-K66N (Fig.   4B). We next performed MS-based immunopeptidome analysis to compare the quality and quantity of peptides bound to the different A*24:02 variants. As shown in Fig. 4(C), compared with WT A*24:02 the A24-G65R mutant demonstrated a ∼50% increase in total number of unique eluted A*24:02-restricted peptides while the A24-K66N mutant showed a dramatic loss of >80% of bound peptides. Comparable numbers of endogenous HLA-I binding peptides were eluted from the same cells, indicating that antigen presentation machinery was functional in all transduced cell lines (Figure S14B, Supplementary Material). As with the A*02:01 variants, peptide repertoire diversity of the A*24:02 variants corresponded well with cell surface levels (Fig. 4C). Also similar to HLA-A*02:01, peptide presentation and surface expression levels of HLA-A*24:02 correlated with the number of basic residues present at HLA-I p65 and p66.

The proportion of D/E4 peptides eluted from the different A24 variants also followed a similar pattern, with A24-G65R binding to a significantly higher percentage of D/E4 peptides and A24-K66N binding a significantly lower frequency of D/E4 peptides compared to A24-WT (Fig. 4D). We next performed peptide binding and stability assays on WT A*24:02 molecules to determine the impact of alterations to peptide position 4. For the strong-binding VYGFVRACL peptide backbone, changing position 4 to D4 or E4 caused a slight increase in stability and a slight reduction in binding affinity, while substituting with A4 caused a reduction in both parameters (Figure S14C, Supplementary Material). Binding assays employing the weak-binding TFSDEAVHF peptide showed that substitution of D4 with G4 also resulted in a loss of binding and stability (Figure S14C, Supplementary Material). These results support the notion that negatively charged residues at p4 enhance HLA-A*24:02/peptide complex stability.

To gain further insights, MD simulations of A*24:02/QFKDNVILL peptide complexes were run for A24-WT, A24-G65R, and A24-K66N (Videos S2, S12, and S13, Supplementary Material). Coulombic interactions and hydrogen bonds were calculated from sampled conformations (Figure S14D, Supplementary Material) and the most prevalent structures were overlaid (Fig. 4E–G). Similar to A24-WT, K66 in the A24-G65R mutant exhibited strong negative coulombic interactions with D4 while R65 showed only weak coulombic interactions with D4 (Figure S14D, Supplementary Material). By contrast, N66 of the A24-K66N mutant exhibited both attractive and repulsive interactions with D4, similar to that observed for N66 in A2-K66N (Figure S10C, Supplementary Material). Alluvial plots revealed salt bridges between D4 and K66 in A24-WT and both A*24:02 mutants, though these were observed to be much less prevalent in A24-K66N (Figure S14D, Supplementary Material). Analysis of overlaid structures illustrated that R65 in A24-G65R interacted primarily with E62 at the top of the binding cleft, thus moving it away from D4 and potentially explaining the limited interactions between R65 and D4 (Fig. 4G). In the A24-G65R mutant, K66 was involved in a network of interactions including with E62 and E63 in addition to peptide residues D4 and F2 (Fig. 4F). In the A24-K66N mutant, N66 showed significantly fewer salt-bridge interactions with the three surrounding acidic residues, but maintained strong hydrogen bond interactions with the backbone of F2 (Fig. 4F). MD simulations with the p4-substituted QFKENVILL peptide complexed to the A*24:02 variants demonstrated similar interactions as the native peptide, with the R65 residue still associated primarily with E62 (Figure S15, Supplementary Material), which notably does not occur in any other classical HLA-I family (Database S1, Supplementary Material).

Since the observed changes in eluted D/E4 peptide frequencies between the different A*24:02 variants did not fully account for the striking changes in peptide repertoire diversity, we next examined the amino acid frequencies at other peptide positions of MS-eluted peptides. This analysis revealed that while peptides eluted from A24-WT and A24-G65R showed very similar characteristics, peptides eluted from A24-K66N exhibited frequency alterations at multiple peptide positions (Fig. 4H; Figure S16A, Supplementary Material). For example, at p1 the K66N substitution resulted in a significant increase in the frequency of K1 peptides (∼15% to ∼20%), although this shift was not nearly as dramatic as that observed for A2-K66N or A2-DM (Figures S16A and S16B, Supplementary Material). Comparing the binding stability of the QFKDNVILL and KFKDNVILL peptides in wet-bench assays showed that the substituted p1 lysine indeed drove increased A*24:02/peptide complex stability (Figure S16C, Supplementary Material). Moreover, peptides eluted from the A24-K66N mutant showed increased frequencies of both dominant A*24:02 anchor residues, Y2 and FΩ with concurrent decreases in the subdominant A*24:02 anchor residues F2, W2, LΩ, and WΩ (Fig. 4I; Figure S16D, Supplementary Material). MD simulations of A*24:02-bound QFKDNVILL, QFKDNVILF, and QYKDNVILL peptides showed that FΩ formed additional Pi ring-stacking interactions with A*24:02 binding-cleft residues Y116 and Y123 compared to LΩ, and Y2 formed additional hydrogen bonds with H70 compared to F2 (Figure S17A, Supplementary Material), providing an explanation for the increased stability and dominance of Y2 and FΩ anchors.

We next investigated the contribution of p4 residues to the binding and stability of A*24:02 peptides with or without dominant primary anchors. The native QFKDNVILL peptide containing two subdominant primary anchor residues (F2 and LΩ), demonstrated relatively poor binding to HLA-A*24:02 (Fig. 4J). Substitution of either primary anchor to Y2 or FΩ notably improved the stability, but altering both anchor positions to Y2 and FΩ greatly improved binding and stability (Fig. 4J). Notably, although D4 to A4 substitution resulted in a loss of peptide stability for all of the primary anchor-altered peptides, the strongest impact was observed in the peptides containing only one dominant primary anchor (Fig. 4K). Consistent with this, MD simulations comparing A*24:02 complexed to QFKDNVILF and QFKANVILF peptides indicated that the D4 to A4 substitution resulted in the loss of a prevalent salt bridge between D4 and K66 (Figure S17B, Supplementary Material). Taken together, these results support that stabilizing interactions between peptide D/E4 residues and K66 of A*24:02 are most critical for peptides that contain subdominant primary anchor residues.

### Peptide position 4 interactions with MHC-I residues are prevalent across the HLA class I system

**Fig. 5.**
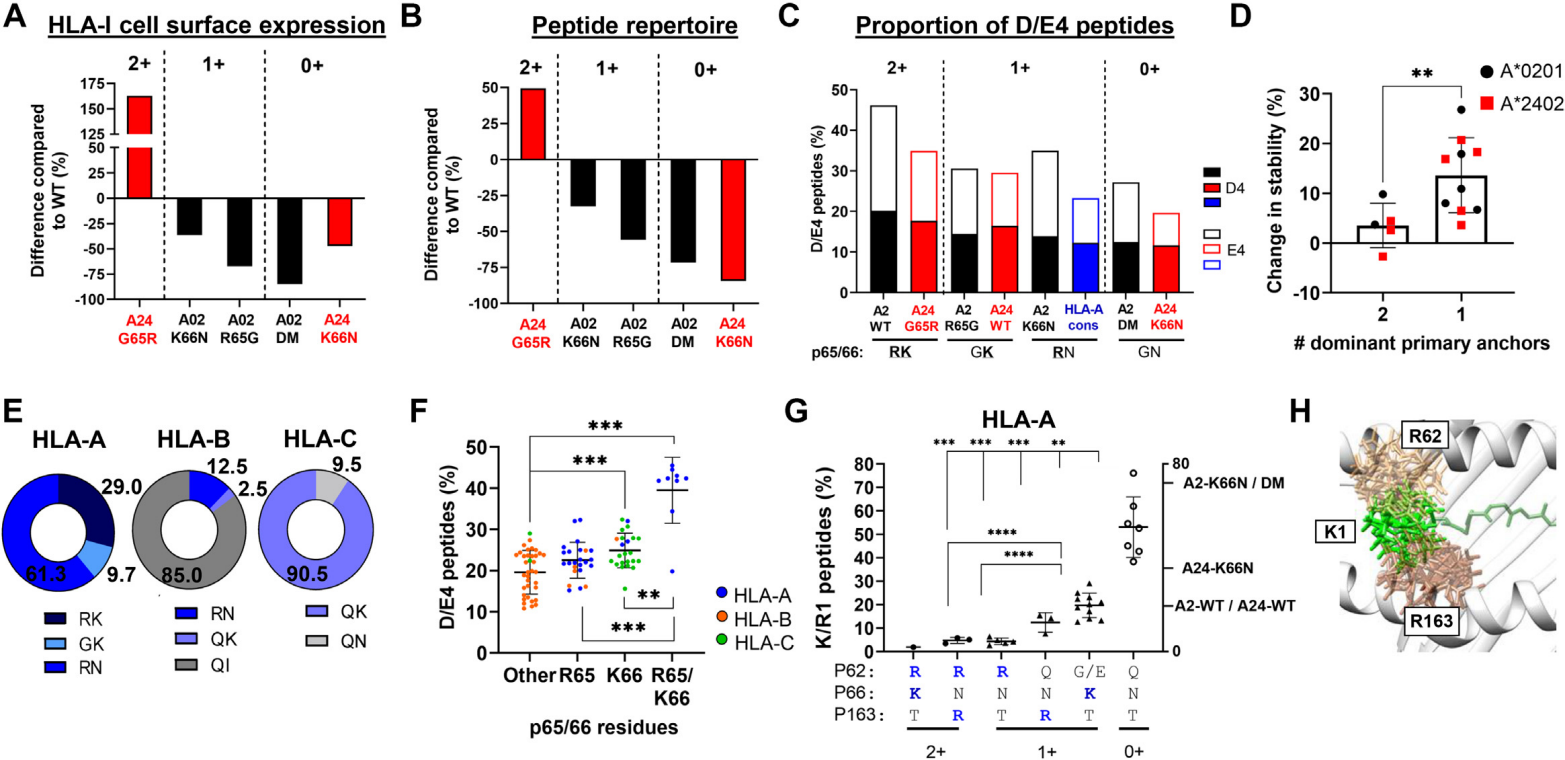
HLA class I/peptide interactions observed in A*02:01 and A*24:02 may be conserved throughout the HLA class I system. (A) Change in cell surface expression between mutated HLA-A*02:01 or A*24:02 molecules compared to their WT counterparts. A*02:01 mutants are shown in black and A*24:02 mutants are depicted in red. (B) Change in the total number of unique peptides identified through MS analysis of mutant A*02:01 and A*24:02 molecules compared to WT A*02:01 or A*24:02 molecules. Numbers at the top of the graph refer to the number of positively charged residues in HLA-I p65 and p66. (C) Comparison of the proportion of D/E4 peptides eluted from A*02:01 or A*24:02 WT and mutated molecules, and other HLA-A allotypes. Black: A*02:01; red: A*24:02; and blue: HLA-A molecules containing the R65/N66 HLA-A consensus sequence. Numbers at the top of the graph refer to the number of positively charged residues in HLA-I p65 and p66. (D) Graph showing the change in peptide stability of A*02:01- and A*24:02-restricted peptides containing D/E4 compared to their non-D/E4 counterparts, with peptides grouped according to the number of dominant anchors (L2-VΩ for A*02:01 and Y2-FΩ for A*24:02).^**^ indicates *P* ≤ 0.01 using an unpaired t test with Welch’s correction. (E) The relative proportions of HLA-A, B, and C allotypes containing R65 and/or K66, where blue color indicates R65 and/or K66 and gray color indicates neither R65 nor K66. (F) The proportion of D/E4 peptide ligands eluted from individual HLA-A, -B, and C allotypes, grouped according to positively charged amino acids in p65 and p66. (G) The proportion of K/R1 peptide ligands eluted from individual HLA-A allotypes, grouped by their configuration of amino acids in HLA-I positions p62, p66, and p163. (H) Computational modeling showing overlap of conformational possibilities between peptide K1 residues (green) and HLA-I residues R62 (light tan) and R163 (dark tan). For (F) and (G), Brown–Forsythe ANOVA tests showed significant difference between means (*P* < 0.0001), and multiple comparisons using Dunnett’s T3 test were also performed to assess the significance of differences between specific groups. ** indicates *P* ≤ 0.01, *** indicates *P* ≤ 0.001, and ^****^ indicates *P* ≤ 0.0001.

In these studies, complementary mutations were made to the p65/66 residues of both A*02:01 and A*24:02 to determine the potential contribution of charged interactions to peptide antigen presentation. For both allotypes, HLA class I molecules containing both R65 and K66 demonstrated the highest cell surface expression and the largest peptide repertoire diversity, while loss of one or both residues resulted in progressive losses of HLA-I surface expression and repertoire diversity (Fig. 5A and B). The K66N substitution was clearly more detrimental to A*24:02 compared to A*02:01, indicating that the R65 residue in A*02:01 may partially compensate for the loss of K66 (Fig. 5B). Comparing the frequencies of eluted D/E4 peptides between the A*02:01 and A*24:02 variants revealed a correlation with the number of positively charged residues present in p65 and p66, supporting our initial hypothesis (Fig. 5C). Binding and stability assays employing panels of synthetic p4 residue-substituted peptides showed that D/E4 residues contribute significantly to increased peptide stability, particularly in weaker-binding peptides containing only one dominant primary anchor residue (*P* < 0.01) (Fig. 5D). These experiments demonstrated that both R65 and K66 make significant contributions to peptide antigen presentation, at least in part by facilitating salt-bridge interactions with D/E4 residues of bound peptide antigens.

Our initial survey of eluted peptide ligands revealed a preference for D/E4 across almost all HLA-I allotypes analyzed (Figure S5, Supplementary Material). All major HLA-A allotypes contain either R65 or K66, and ∼30% of them possess both residues—all from the HLA-A2 family with one exception, HLA-A*34:01 (Fig. 5E; Database S1, Supplementary Material). K66 is the consensus amino acid in HLA-C allotypes, being present in all except for HLA-C*07:01 and C*15:02, which both possess N66. By contrast, K66 is found in only one HLA-B allotype (HLA-B*46:01), and R65 is present in only five out of 36 HLA-B allotypes analyzed (Fig. 5E) In HLA-Bs, isoleucine (I) is the p66 consensus amino acid and glutamine (Q) is found at p65 of nearly all HLA-B and -C allotypes (Fig. 5E). HLA-I allotypes possessing either R65 or K66 bound to a higher proportion of D/E4 peptides compared to allotypes with no positively charged amino acids at these p65/66 positions. As expected, HLA-I molecules harboring both R65 and K66 presented the highest proportion of D/E4 peptide ligands (*P* < 0.001; Fig. 5F). Collectively, these results show that D/E4 peptide ligand binding preferences correlate with the presence of R65/K66 across the HLA-I system. Furthermore, positively charged residues at p65/66 are observed in several animal MHC class I allotypes (Table S3, Supplementary Material) that also prefer D/E residues at peptide position 4 (Figure S1B, Supplementary Material), indicating conservation of this interaction across species.

The dramatic increase observed in the frequency of K/R1 peptide ligands eluted from both A2-K66N and A2-DM was an unexpected finding that led us to further explore potential interactions involving this p1 residue. Analysis of peptide ligands eluted from HLA-A, -B, and -C molecules revealed a wide diversity of preferences for K/R1 peptides among class I allotypes, with frequencies ranging from <1% to >75%. (Database S1, Supplementary Material). Although N66 is the consensus residue found in most HLA-A allotypes, only a subset of these demonstrated a clear preference for K/R1 peptide ligands (Fig. 5G). Examination of different HLA-I/peptide crystal structures revealed that HLA-I residues p62 and p163 are typically in close proximity to peptide position 1 residues. Based on this, we grouped HLA-A, -B, -C, and -G allotypes according to their respective amino acid configurations of p62, p66, and p163 and then assessed the preferences of each group for binding K/R1 peptides (Figures S18A–S18C, Supplementary Material). As shown in Fig. 5(G), the nine HLA-A allotypes containing R62 all presented an extremely low frequency of K/R1 peptides (<3%). By contrast, HLA-A allotypes featuring R163 or K66 bound to K/R1 peptides at frequencies of 12% and 20%, respectively. However, the seven HLA-A allotypes lacking all three basic residues R62, K66, and R163 presented K/R1 peptides at a significantly elevated mean frequency of ∼50% ( *P* < 0.01). Notably, similar trends were also observed for HLA-B, -C, and -G molecules (Figure S18B and S18C, Supplementary Material).

To gain molecular insights into these binding preferences, we performed rotamer library analysis of the p1-substituted KLKEGIPAL peptide bound to A*02:01 containing G62R or T163R substitutions. As shown in Fig. 5(H), this analysis revealed significant overlap between the possible states of these large side chains. By contrast, such overlap between residues was not observed in the presence of smaller amino acids alanine or serine at p1 (Figure S18D, Supplementary Material). MD simulations further confirmed repulsive interactions between K1 and K66 (Figure S18E, Supplementary Material). Collectively, these data support the notion that K/R1 peptide binding preferences of HLA-I molecules are largely dictated by particular amino acid configurations at p62, p66, and p163 that are capable of sterically accommodating large p1 side chains.

## Discussion

The dominant contributions to HLA-I binding made by B and F pocket-occupying residues found at the p2 and pΩ positions of peptide ligands is well-established (45–52), but the impact of other N-terminal peptide residues on antigen presentation has remained largely undescribed. Our analysis of two prevalent HLA-I allotypes, A*02:01 and A*24:02, provides evidence that HLA-I/peptide interactions involving p1 and p4 play a significant role in peptide-binding stability and can strongly influence overall antigen presentation as analyzed at the immunopeptidome level. The preponderance of negatively charged p4 residues in peptide ligands eluted from across the HLA-I system suggested that they may play a subdominant, “pan-anchor” role; furthermore, HLA-I/peptide crystal structures revealed the potential for charged interactions with two positively charged HLA-I residues at p65/66 found in close proximity to p4 near the top of the peptide-binding groove (48, 53–55). Our mutational analysis demonstrated that these p65/66 residues were required for replete HLA-I cell surface expression and antigen presentation, and their removal resulted in a dramatic reduction in the diversity of presented peptides. Binding and stability assays revealed that peptide D/E4 residues do make significant contributions to the stabilization of A*02:01 and A*24:02 complexes, and that this interaction was most critical for peptides with weaker or subdominant primary anchors at p2 and pΩ. Since peptide binding to MHC-I in the endoplasmic reticulum (ER) is a prerequisite for transport to the cell surface via the secretory pathway, we speculate that the reduction in peptide repertoire binding diversity may limit the rate of production of stable HLA-I/peptide/β2m complexes in the ER. Since the p65 and p66 amino acid substitutions that we made are found naturally in several other common HLA-I allotypes, we believe it is unlikely that they had a negative impact on fundamental HLA-I biogenesis, including folding and interactions with ER chaperones. However, future experiments to delineate the precise dynamics of antigen loading and intracellular trafficking in the HLA-I mutants will be required to fully resolve these questions.

Our results suggest that although R65 and K66 demonstrate some overlapping roles, they appear to make uniquely distinct contributions to peptide binding. MD simulations showed that K66, located on the inner side of the binding cleft, forms a network of interactions with multiple peptide positions (p1, p2, and p4) and multiple HLA-I residues (p62, p63, p65, and p70), many of which were disrupted with the N66 substitution. R65, by contrast, is located at the top of the *α*1 helix and was observed to interact directly only with p4 and the HLA-I residues p62 and p66. Nonetheless, removal of R65 from A*02:01 resulted in a >50% reduction in HLA-I expression and peptide repertoire diversity, and also resulted in alterations to the C-terminal anchors of eluted peptides, consistent with a role for R65 in facilitating peptide stability. In a recent study simulating the unbinding pathways for A*24:02-restricted peptides, K66 was shown to help prevent the unbinding of peptide N-termini, even while pΩ was already completely detached (39). Considering the broad impact of both R65 and K66 mutations on antigen presentation described herein, we speculate that these residues may act in part as “gatekeepers” for peptide unbinding. Further studies are needed to confirm this hypothesis and to evaluate whether R65/K66 might have been selected to promote the stability of MHC-I/peptide complexes and overall peptide diversity, as their evolutionary conservation within subsets of MHC-I allotypes would suggest.

One of the more surprising findings of our study was the starkly different impacts that the K66N substitution had on HLA-A*02:01 and A*24:02. Although K66 loss in A*24:02 resulted in a dramatic decrease in surface expression and peptide diversity, peptide N-terminal amino acid frequencies showed minimal alterations. By contrast, K66N substitution of A*02:01 in both the A2-K66N and A2-DM mutants resulted in a striking increase in the frequency of positively charged amino acids arginine or lysine in position 1 of eluted peptides. A similar phenotype was previously reported for peptides eluted from the naturally occurring A*02:20 allotype (which is identical to A2-K66N), in accordance with our findings (21). Our survey of 108 major HLA-I allotypes showed that only those allotypes lacking R62, K66, and R163 residues strongly favor the binding and presentation of K/R1 peptides; consistent with this, molecular modeling demonstrated that these three HLA-I residues have the potential to cause both steric hindrance and charge repulsion of the long side chains of K/R1 residues, as had been previously proposed (21). We also noted an intriguing positive correlation in many different HLA-I allotypes between the occurrence of K/R1 and D/E4 in eluted peptide ligands. Intrapeptide interactions, including those between p1 and p4, have previously been proposed to contribute to the binding affinity and immunogenicity of specific peptides (31, 53, 56, 57). In accordance with this, our MD simulations showed the potential for an intrapeptide salt bridge between K1 and D/E4. Taken together, these results suggest a more general role for ionic intrapeptide interactions between positions 1 and 4 in facilitating HLA-I/peptide stability and overall antigen presentation.

It is important to note that a number of studies have addressed the importance of R65 and K66 for TCR engagement and CD8+ T-cell activation (58–60). TCRs recognize structural and biochemical features, especially electrostatic potential, across the combined surface of HLA-I/peptide complexes. In fact, negatively charged residues at p4 of peptide ligands have been implicated in disrupting T-cell recognition (61, 62), and in driving the clonality and specificity of CD8+ T-cell responses (63, 64). Moreover, a recent computational study identified p4 as the most important position for determining peptide immunogenicity (29). In light of our results showing the prevalence of salt-bridge interactions mediated by R65/K66, we postulate a dual role for these residues in determining both peptide binding stability and shared surface patterns available for TCR recognition (Figure S19, Supplementary Material). Considering the known inverse complementarity of electrostatic potential between TCRs and cognate MHC-I/peptide surfaces, it is conceivable that charged surface patterns involving HLA-I p65/66 and peptide D/E4 residues may constitute “hot-spots” recognized by public TCRs (59). In support of this idea, previous studies have found that human A*02:01-restricted TCRs are enriched for the capacity to engage with a positively charged hot-spot involving R65 (65, 66). Such precise charge complementarity would be important to facilitate TCR engagement with A*02:01-restricted complexes due to the high energetic cost associated with desolvation of charged residues. The cost of desolvation has been used to predict that peptides with charged residues may be less immunogenic, especially when involving central peptide positions (67). However, our results raise the possibility that peptide D/E4 residues might contribute to reducing the desolvation cost associated with R65/K66, thereby facilitating TCR recognition.

The findings reported here advance our fundamental understanding of peptide ligand binding and MHC-I-mediated antigen presentation and raise several implications. For example, peptide ligands restricted to HLA-A*02:01 and A*24:02 that contain D/E in peptide position 4 could be of particular interest as targets for vaccines or T cell-based immunotherapies against viral diseases or cancer, especially for peptides with subdominant primary anchors. This same rationale may also apply to other HLA-I allotypes containing R65 and/or K66, although further studies will be required to confirm this. Based on their frequent co-occurrence throughout the HLA-I system, ligands containing both K/R1 and D/E4 may show further enhanced stability in the binding cleft, as the intrapeptide interactions between them would suggest; it will be of great interest to assess how TCR binding might impact these interactions. More broadly, the rules uncovered by this study may be leveraged to improve peptide binding predictions for the many HLA-I allotypes lacking well-defined binding motifs. Furthermore, it is probable that other shared MHC-I/peptide interactions exist throughout the HLA-I system that remain to be identified. The approach used here, combining site-directed mutagenesis, global immunopeptidome analysis, quantitative wet-bench assays, and computational simulations provides a road map for discovering such interactions, which will ultimately facilitate improved accuracy of immune targeting in many different human disease contexts.

## Materials and Methods

### Crystal structures and visualization

Crystal structures of selected HLA-I/peptide complexes were obtained from the Protein Data Bank (PDB, 55) and minimally revised using the PyMOL Viewer (68) to make images of crystallographic structures. Further processing was conducted on crystal structures used as input for MD (see below). Cartoon representations of the MHC-Is, cross-section views of the binding clefts, and side view images of the peptides were obtained with the UCSF Chimera (69) and UCSF ChimeraX packages (70). These packages are developed by the Resource for Biocomputing, Visualization, and Informatics at the University of California, San Francisco (supported by NIGMS P41-GM103311 and R01-GM129325).

### Modeled structures of HLA-I/peptide complexes

Additional HLA-I/peptide structures were modeled to account for peptide variants or HLA-I mutations of interest to our study, and for which no crystal structures were available on PDB. These additional structures mostly related to single amino acid substitutions, or very limited combinations of substitutions, in relation to an available reference crystal structure. These substitutions were performed with the mutagenesis wizard of PyMOL Viewer, based on the best ranked rotamer for each substitution. Note that these modified HLA-I/peptide structures were used as input for MD (i.e. full-atom, explicit solvent, and molecular mechanics simulation), which should further resolve any issues related to suboptimal positioning of the mutated amino acids.

### MD simulations and analysis

Input structures for MD simulations were preprocessed with the PDB2PQ webserver (71). Structures were fixed as needed, protonated at pH 7.0 using the PROPKA algorithm (72), and atom distances and hydrogen bonding networks were optimized according to the CHARMM force field. All MD simulations were performed with the GROMACS 2021 package (73) using the CHARMM36 force field (74) and the TIP3 water model. Preprocessing steps of Energy Minimization and Equilibration were conducted as previously described (75, 76). The production stage consisted of five independent simulations of 20 ns for each complex of interest, for a combined sampling of 100 ns per complex, with better thermodynamical descriptors (77–79). Analysis was conducted with Python and R packages including MDtraj (80), GetContacts (https://getcontacts.github.io/), and ggplot2 (81). Additional details are provided in the Supplementary Methods.

### Cell lines and generation of HLA-A*02:01 and HLA-A*24:02 mutant alleles

H1975 lung cancer cells were purchased from the ATCC (CRL-5908). Site-directed mutagenesis using the QuikChange II site-directed mutagenesis kit (Agilent, 200521) was performed on WT HLA-A*02:01 and HLA-A*24:02 cDNAs subcloned into pDONR221 (ThermoFisher Sceintific, 12536017). Creation of the A*02:01 (R65G, K66N, and R65G/K66N) and A*24:02 (G65R and K66N) mutant constructs were confirmed by Sanger sequencing. WT and mutated A*02:01 and A*24:02 cDNAs were subcloned into destination lentiviral expression vector pLV460 using LR reaction-based methodology (ThermoFisher Scientific, 11791020).

### Lentiviral production and transduction

293METR cells were plated in 10 cm plates (Corning, 430167) and transfected with 10 μg pCMVdelta8.91, 1 μg VSV-g, 10 μg pLV460 with A*02:01 or A*24:02 variant cDNAs, and 50 μl lipofectamine 2000 (ThermoFisher Scientific, 11668019). Viral supernatant was collected after 2 and 3 days, centrifuged and concentrated using Lenti-X (Takara BioSciences, 631232). The lung cancer cell line H1975 (which expresses the endogenous HLA-I allotypes HLA-A*01:01, B*41:01, and C*17:01) was transduced with lentiviral vectors expressing WT or mutated HLA-A*02:01 or HLA-A*24:02 in 4 μg/ml polybrene (Millipore Sigma, TR-1003-G), and surface expression was analyzed by staining with HLA-A*24:02-specific (17A10, MBL International, K0208-3) or HLA-A*02:01-specific (BB7.2, BioLegend, 343308) antibodies and flow cytometric analysis (BD FACS Canto II).

### HLA class I immunoprecipitation and peptide elution

Peptides were eluted from immunoprecipitated HLA-I molecules as described previously (82). In brief, transduced or untransduced H1975 cell lines were expanded in 10 cm plates (Corning, 430599), lysed using Triton X-100 (Sigma Aldrich, T9284), and incubated overnight at 4°C under gentle agitation with 50 μg of the pan HLA-A, B, C-specific antibody W6/32 for every 10 mg of protein. HLA class I molecules were immunoprecipitated by using Protein A/G UltraLink resin beads (ThermoFisher Scientific, 53133) and then directly eluted on columns (ThermoFisher Scientific, 89897) using 0.1 N acetic acid (Fisher Scientific, BP2401). HLA-I purification was confirmed by Western blot and eluted fractions were pooled prior to LC-MS/MS analysis.

### LC-MS/MS analysis of eluted peptides

Peptides eluted from HLA-I molecules on H1975 cells were desalted by Zip-Tip, and split in half with one half analyzed by the Proteomics Facility at U.T. MD Anderson Cancer Center and the other half analyzed by the ThermoFisher facility in San Jose, CA. Analysis of peptides eluted from HLA-I molecules at the Proteomics Facility at U.T. MD Anderson Cancer Center was similar to a previous report (82). In brief, peptides were analyzed by LC-MS/MS using a Nano-cap LC system (Dionex U3000) attached to a tribrid mass spectrometer (Orbitrap Fusion, Thermo Scientific). Chromatography was performed in a 25 cm × 75 um column packed with 2 micron C18 particles (col) using 0.1% formic acid in water as solvent A and 0.1% formic acid in acetonitrile as solvent B. MS/MS was performed using the linear ion trap and ESI where the full MS1 range was 265 to 1,500 m/z for singly charged precursors, 400 to 750 m/z for doubly charged precursors, and 270 to 500 m/z for triply charged precursors. Spectra acquired by LC-MS/MS was analyzed using MASCOT (v2.6, Matrix Science, London) to search spectra against the SWISSPROT human proteome database. Peptide elutions performed at the ThermoFisher Scientific facility in San Jose, CA were separated using an EASY nLC 1200 System (Thermo Scientific) equipped with an EASY-Spray Emitter. MS was performed on an Exploris 480 quadrupole-Orbitrap mass spectrometer (Thermo Scientific) with the FAIMS Pro ion-mobility attachment. Nano-LC was performed on a 75um × 25 cm C18 column and eluted with 0.1% formic acid in water/acetonitrile essentially as described above.

### Quality control of mass spectrometry-identified peptides

Individual peptide matches underwent quality assessment by reference to multiple orthogonal parameters, including Mascot Ion score (minimum score 25), MS1 measured differential to the calculated peptide mass (delta mass), and predicted binding to the relevant HLA-I allotypes as determined by high-resolution genetic sequencing and the NetMHCpan4.1 algorithm (15). Peptide matches were analyzed by BLAST searches to identify all potential source genes, which were then cross-referenced to RNAseq data derived from parental H1975 cells to provide further validation of peptide identity (validation requiring a minimum source gene expression of 0.3 transcripts per million, TPM). All unique peptides identified from multiple MS runs that passed quality control were assigned to an individual HLA-I allotype, which was enabled by the fact that the endogenous and transduced HLA-I allotypes showed sufficiently distinct peptide binding motifs (Figure S20, Supplementary Material).

### Peptide binding and stability assays

All HLA-A*02:01- and A*24:02-restricted peptides used in the study were purchased from Genscript at purity of > 80% with quality control by reverse-phase HPLC and mass spectrometry (SC1208). Recombinant HLA-A*02:01 and HLA-A*24:02 molecules were made and re-folded as described elsewhere (83). Conformation-dependent W6/32 antibodies used in these assays were produced in vitro from hybridoma cell lines (Integra, Chur, Switzerland). All A*02:01 and A*24:02 binding assays were done as described previously (83). In brief, streptavidin donor beads (PerkinElmer, 6760002) and acceptor beads (PerkinElmer, 6762001) conjugated to W6/32 were incubated with A*02:01 or A*24:02 that had been preincubated with varying peptide concentrations. Plates were read by PerkinElmer EnVision and data was analyzed using GraphPad Prism. Peptide stability assays were performed as described previously (84). In brief, biotinylated A*02:01 and A*24:02 molecules were refolded with excess peptide then incubated with 0, 2, 4, or 6 M urea. Nondenatured peptides were detected by W6/32 staining using ELISA as described previously (85). Stability was calculated as a percentage relative to a reference known binder peptide with an assigned stability index of 100%. LLFGYPVYV was used as the reference peptide for A*02:01 and TYTQDFNKF was used as the reference peptide for A*24:02 in both the binding and stability assays.

### Statistical analysis

Differences between WT and mutated amino acid positional frequencies of eluted peptides were analyzed using the 2-sample test (Z test) for equality of proportions without continuity correction. Comparisons of two means with unequal variances were analyzed using unpaired t tests with Welch’s correction. Comparisons of multiple means with unequal variances were performed using Brown–Forsythe ANOVA and multiple pairwise comparisons were then performed using Dunnett’s T3 test.

## Supplementary Material

pgac124_Supplemental_Files

## Data Availability

Raw mass spectrometry data from all HLA class I peptide elutions will be deposited to the ProteomeXchange Consortium database via the publically available PRIDE partner repository.
