# Supplementary material for: Charge-based interactions through peptide position 4 drive diversity of antigen presentation by human leukocyte antigen class I molecules
Source: PNAS Nexus. 2022 Jul 27;1(3):pgac124. doi: 10.1093/pnasnexus/pgac124 (PMC9391200; doi:10.1093/pnasnexus/pgac124)
Supplement: pgac124_Supplemental_Files [file pgac124_supplemental_files.zip › SUPPLEMENTARY FIGURES.pdf]

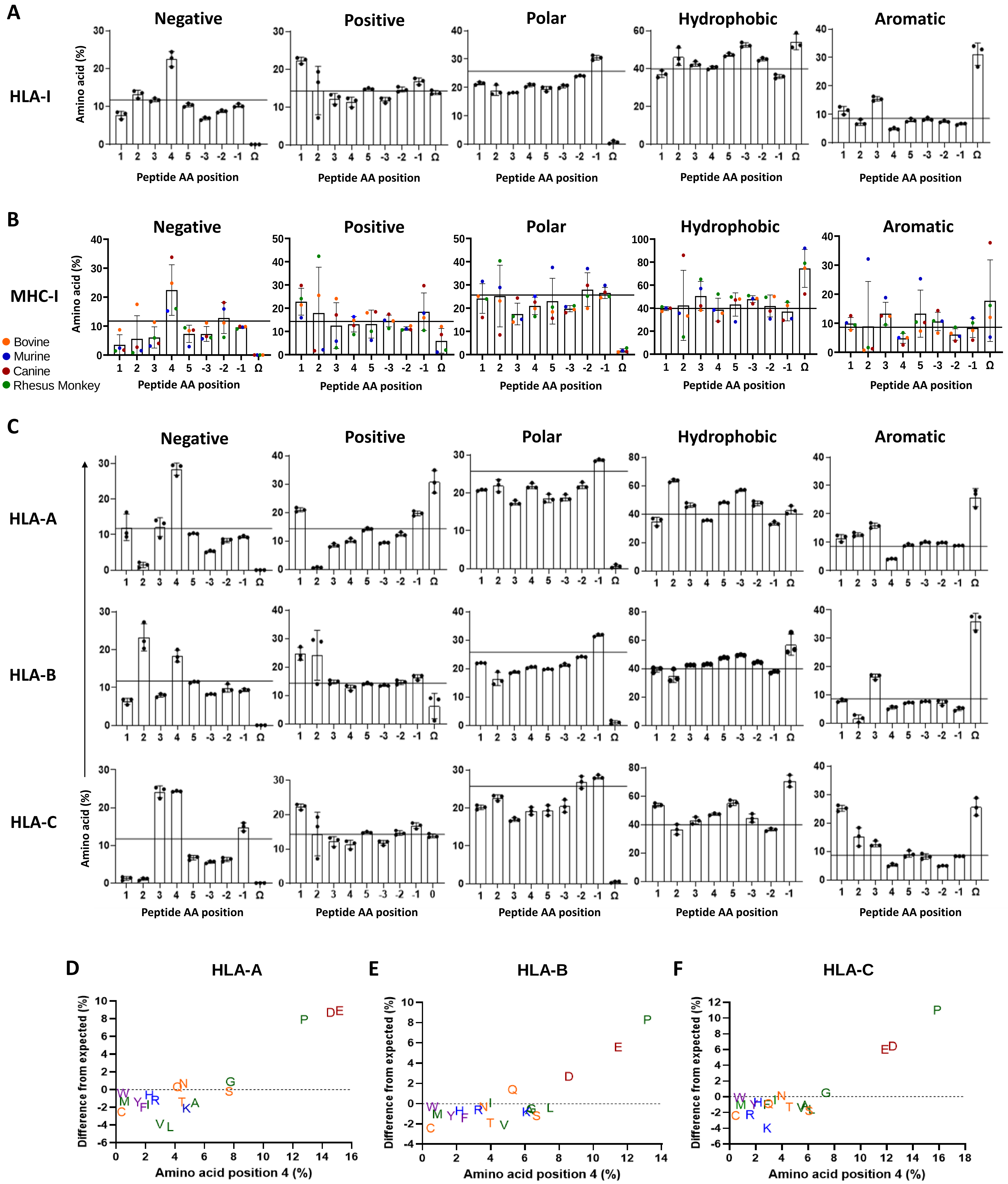

Figure S1. Amino acid content of MHC class I peptide ligands eluted by mass spectrometry: **(A)** Survey of mass spectrometry (MS)-eluted peptides from 108 HLA class I allotypes, showing the frequency of amino acids with shared biochemical characteristics at different peptide positions. Amino acid groupings include those with negatively charged (D, E), positively charged (K, R, H), polar (C, N, Q, S, T), hydrophobic (P, A, G, I, L, M, V), or aromatic (F, W, Y) side chains. Each dot represents one of 3 public peptide ligand databases queried: MS-eluted peptides from the Immune Epitope Database (IEDB), the MHCFlurry training dataset, and the training dataset used for HLAthena. Black horizontal lines depict the background frequencies of each amino acid group in vertebrate proteins. To accommodate peptides of different lengths, indicated peptide positions were numbered starting from the N-terminus (1 to 5) or from the C-terminus (Ω to -3). **(B)** Survey of peptides eluted from MHC class I molecules of 4 different animal species, analyzed as described in (A). Individual dots depict peptide frequencies eluted from bovine, murine, canine or rhesus monkey MHC-I molecules. **(C)** Similar survey analysis as in (A), but data has been separated into HLA-A, -B, or C classes. **(D-F)** Comparison of amino acid prevalence at position 4 of peptides eluted from HLA-A, -B, or -C allotypes. The x-axis shows the actual frequencies, and the y-axis indicates the difference compared to the background frequency of each amino acid in vertebrates. AA, amino acid.

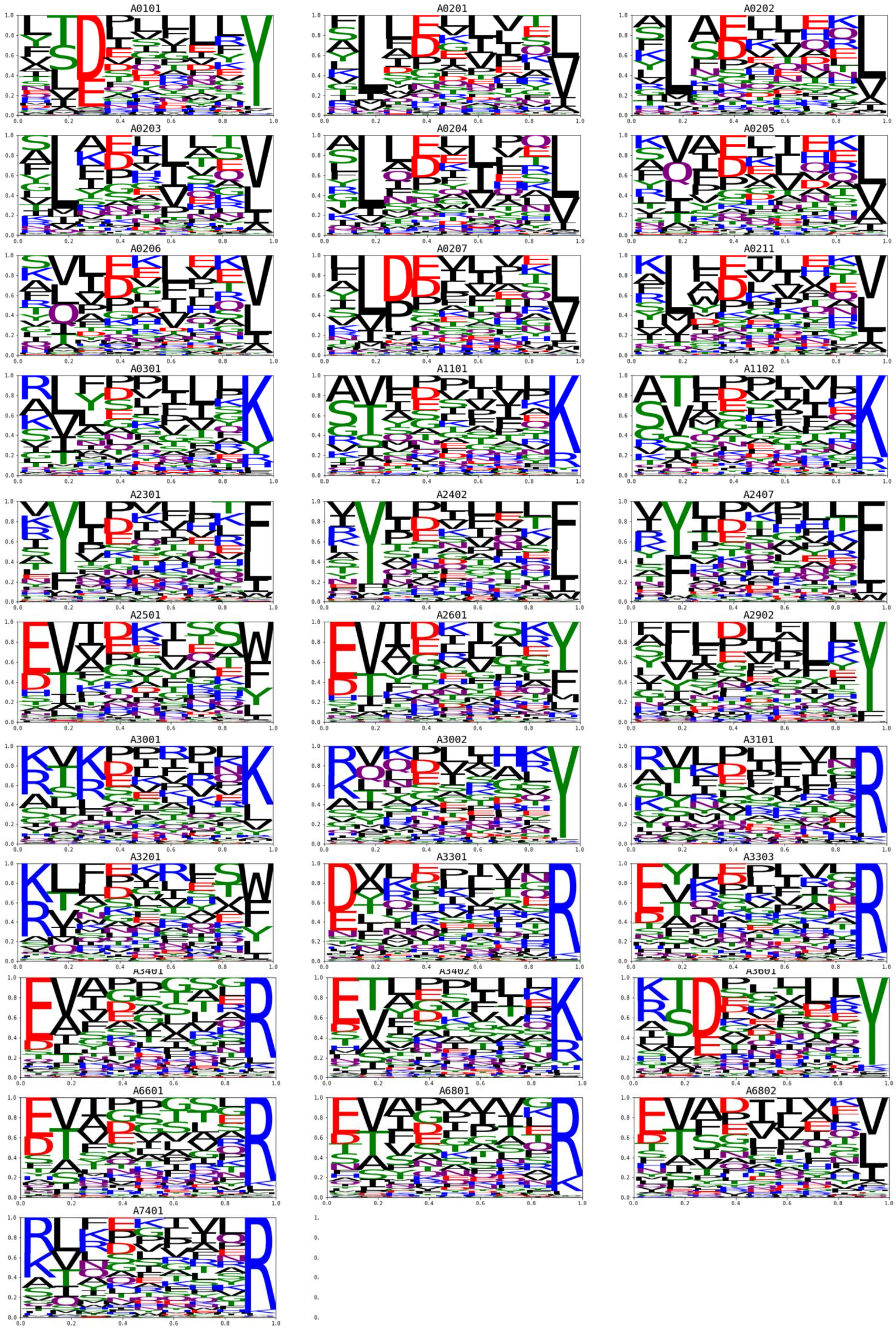

Figure S2. Amino acid frequencies by position in peptides bound to HLA-A allotypes: True logo representations of amino acid frequencies at all peptide positions, derived from 31 HLA-A allotypes. The one-letter codes representing each amino acid are proportional in size to the residue frequency at that position and are listed from the top in descending order of prevalence. To accommodate peptides of different lengths, the first 5 peptide positions were counted from the peptide N-termini, and the last 4 peptide positions were counted from the C-termini. MS-eluted peptides (8 to 11-mers) from the HLAthena training dataset were used to generate logo visualizations.

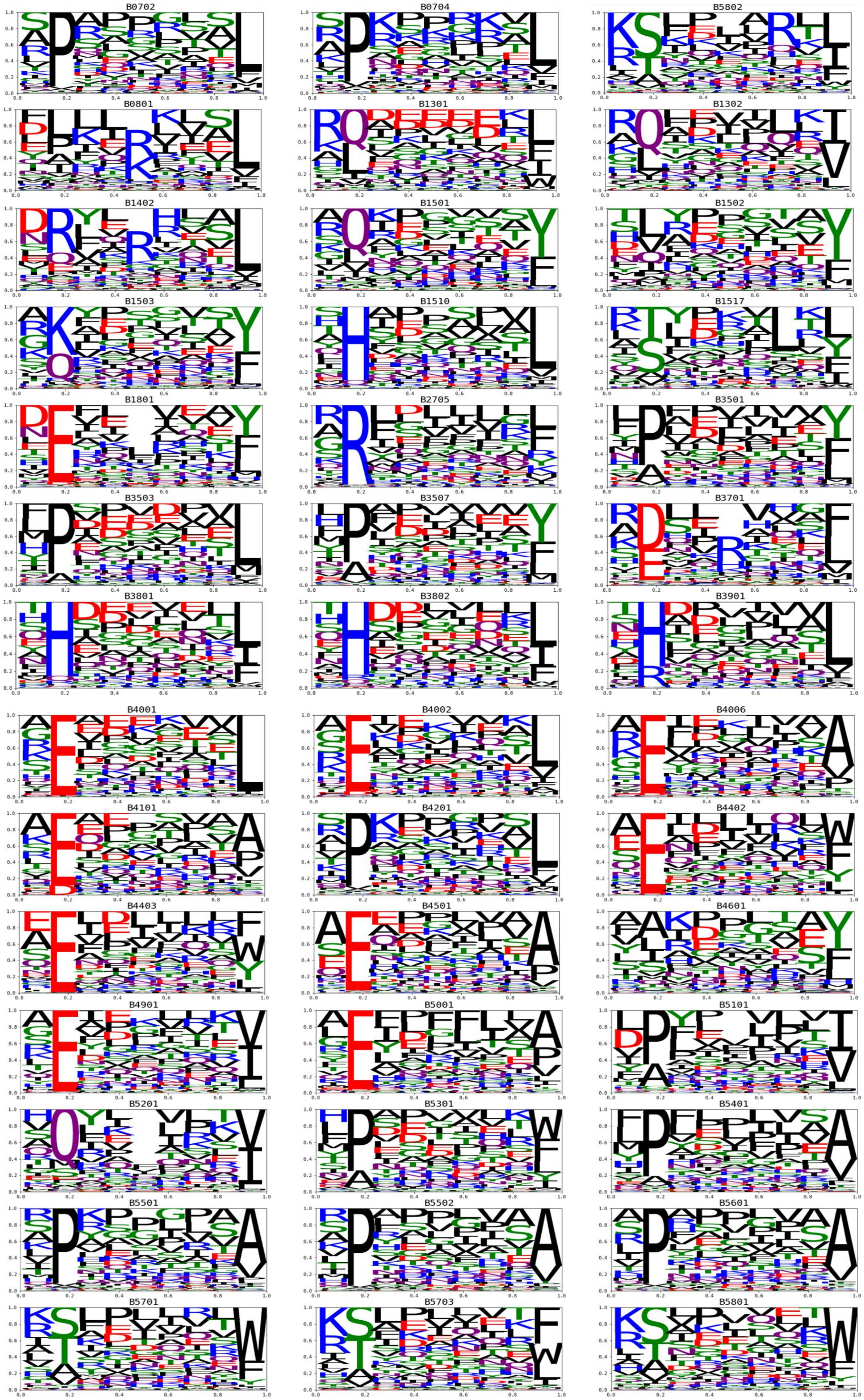

Figure S3. Amino acid frequencies by position in peptides bound to HLA-B allotypes: True logo representations of amino acid frequencies at all peptide positions, derived from 42 HLA-B allotypes. The one-letter codes representing each amino acid are proportional in size to the residue frequency at that position and are listed from the top in descending order of prevalence. To accommodate peptides of different lengths, the first 5 peptide positions were counted from the peptide N-termini, and the last 4 peptide positions were counted from the C-termini. MS-eluted peptides (8 to 11-mers) from the HLAthena training dataset were used to generate logo visualizations.

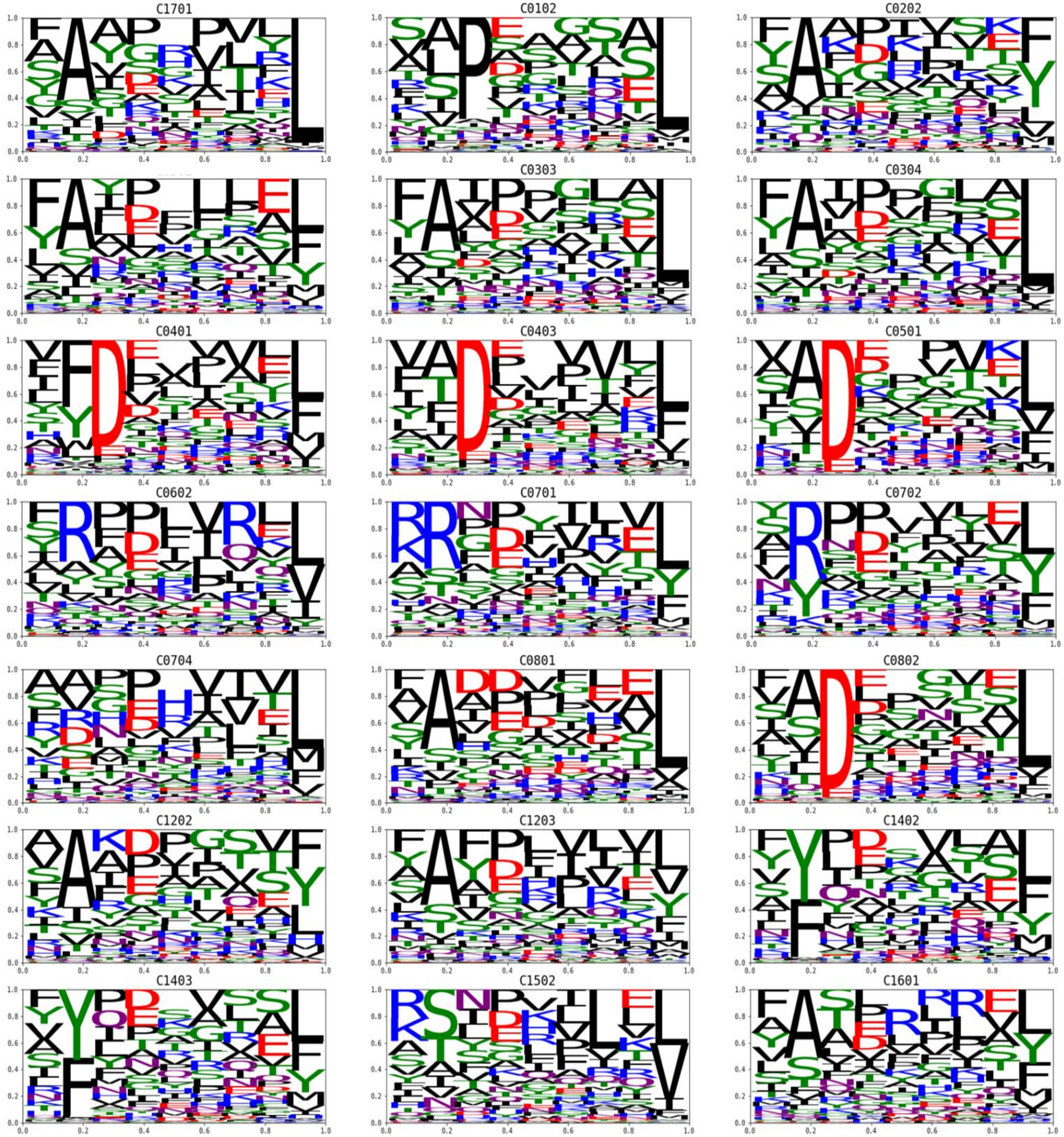

Figure S4. Amino acid frequencies by position in peptides bound to HLA-C allotypes: True logo representations of amino acid frequencies at all peptide positions, derived from 21 HLA-C allotypes. The one-letter codes representing each amino acid are proportional in size to the residue frequency at that position and are listed from the top in descending order of prevalence. To accommodate peptides of different lengths, the first 5 peptide positions were counted from the peptide N-termini, and the last 4 peptide positions were counted from the C-termini. MS-eluted peptides (8 to 11-mers) from the HLAthena training dataset were used to generate logo visualizations.

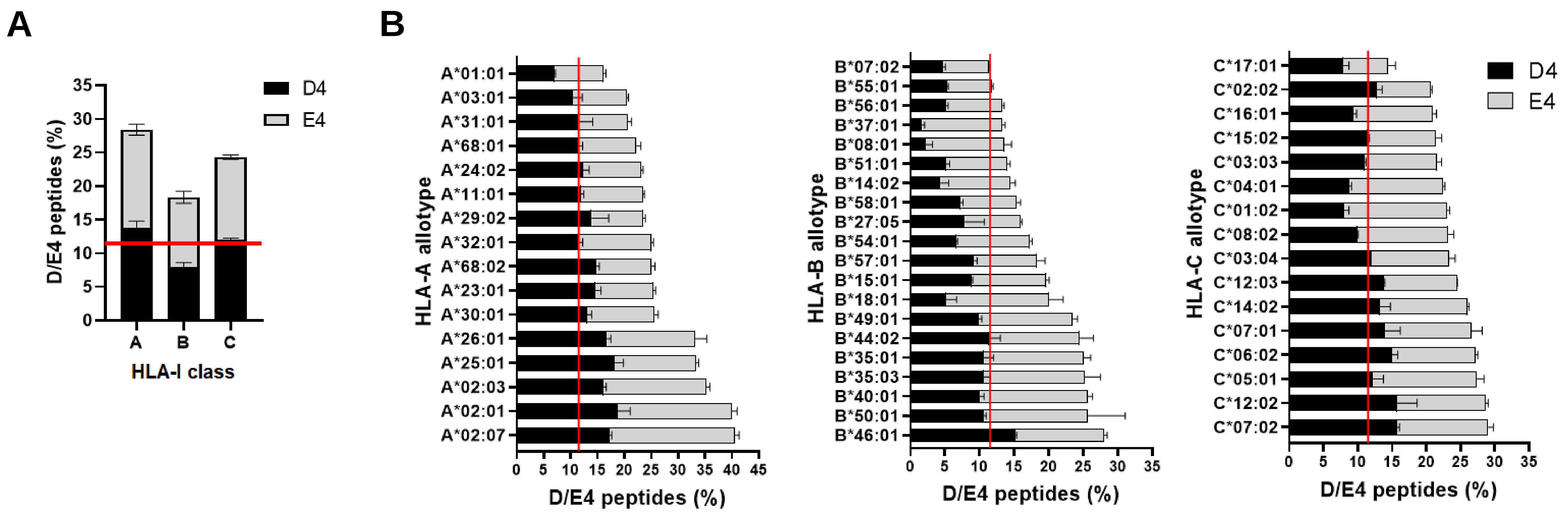

Figure S5. D/E residues are highly preferred at position 4 of peptides eluted from most HLA-A, B and C allotypes: (A) Frequency of eluted peptides containing D4 or E4 in HLA-A, B or C allotypes from 3 peptide databases, with the red line indicating the background D/E4 frequency. **(B)** Proportion of D/E4 peptides eluted from multiple HLA-A, B and C allotypes. Error bars indicate the use of three public HLA-I peptide ligand databases. The red line indicates the combined background frequency of D/E amino acid residues in vertebrate proteins (~12%).

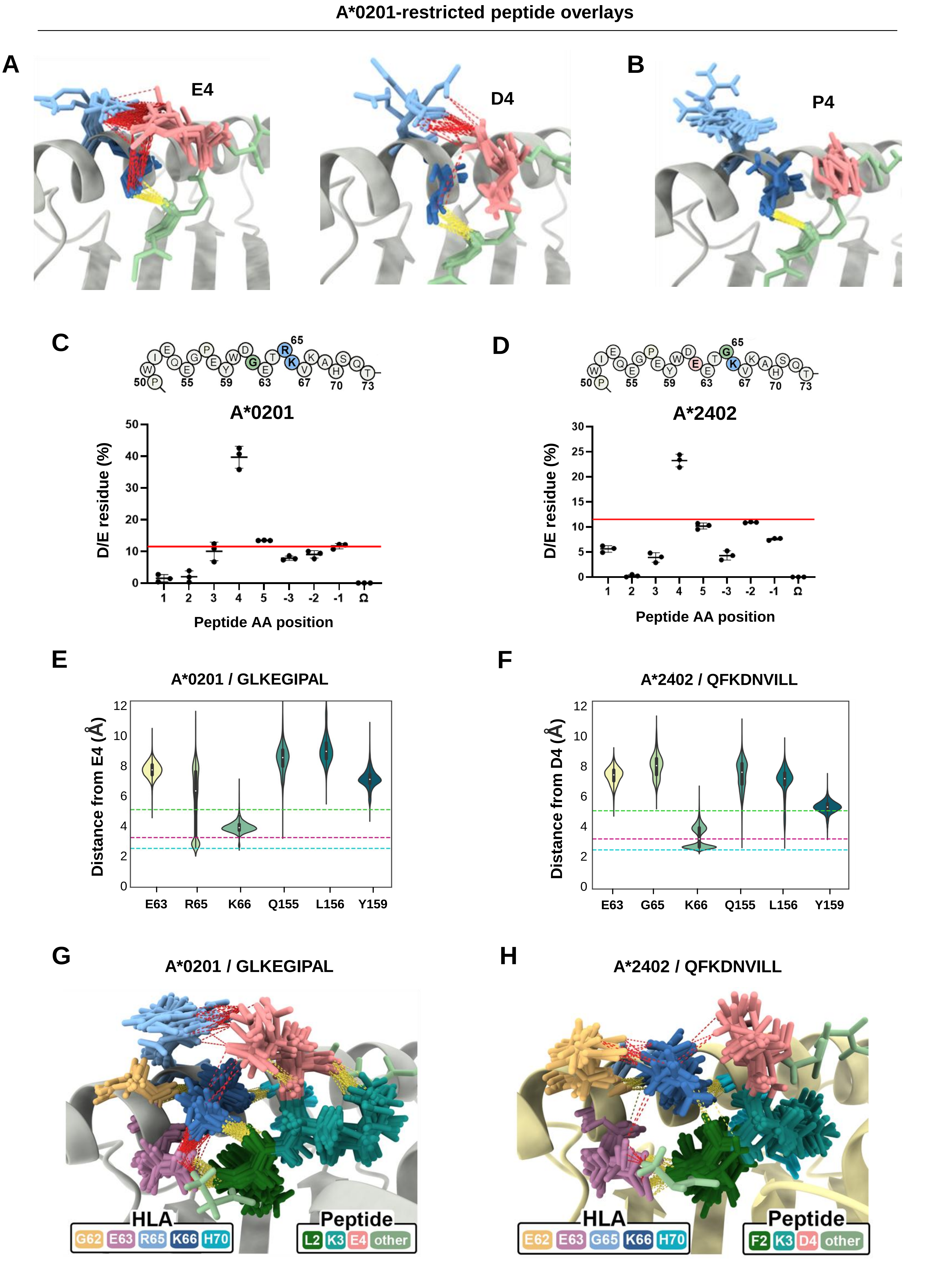

Figure S6. Negatively charged residues in peptide position 4 can interact with R65 and K66 of HLA class I molecules: **(A-B)** Overlap of multiple crystal structures of HLA-A*0201-restricted peptides containing either E4 (A, left), D4 (A, right) or P4 (B). Alternative conformations of the side chains of peptide residue p4 (red), as well as A*0201 residues p65 (light blue) and p66 (dark blue) are displayed as sticks. Multiple backbone conformations for peptide position 2 are also displayed as sticks, while for the other peptide positions only the main chain atoms of a single conformation are depicted (green). For other A*0201 residues, only the main chain of a single conformation is depicted. The crystal structures depicted here include examples of peptides containing D4 (6TRO, 6TRN, 6AMU, 6AMT, 3FT4, 3FT3, 1I4F), E4 (6VRM, 6VR5, 6VR1, 5HHP, 5HHO, 5F7D, 5ENW, 5DDH, 1I1Y, 1I1F, 1AKJ) or P4 (6Q3K, 5FDW, 5FA4, 5FA3. 5F9J, 5EU6, 5EU5, 5EU4, 5EU3, 5EOT, 5D2N, 5D2N, 5D2L, 4WUU, 3MRD, 3MRC, 3MRB, 3MR9, 3HPJ, 3GSX, 3GSW, 3GSV, 3GSU, 3GSR, 3GSQ, 3GSO, 2X4T, 2X4R, 2X4O). **(C-D)** Amino acid sequences of HLA-I positions 50-73 from the α1 helices of A*0201 (C) and A*2402 (D), along with the frequency of D/E residues by position in peptides eluted from each allotype in 3 different peptide ligand databases. **(E-F)** Violin plots depicting the distances between peptide p4 (D or E) and key A*0201 or A*2402 residues across all conformations extracted from molecular dynamics (MD) simulations of the same HLA-I/peptide complexes (see Methods). **(G-H)**. Ensemble of representative conformations extracted from multiple molecular dynamics (MD) simulations of (G) the GLKEGIPAL peptide bound to WT A*0201, or (H) the QFKDNVILL peptide bound to WT A*2402. Alternative conformations of the side chains of peptide residues 2, 3 and 4, as well as HLA-I residues 62, 63, 65, 66 and 70 are depicted as sticks and colored by amino acid position as indicated by the legend. For other peptide positions, only the main chain atoms of a single conformation are depicted. For other HLA-I residues, only the main chain of a single conformation is depicted. Yellow dashed lines indicate hydrogen bonds and red dashed lines indicate salt bridges.

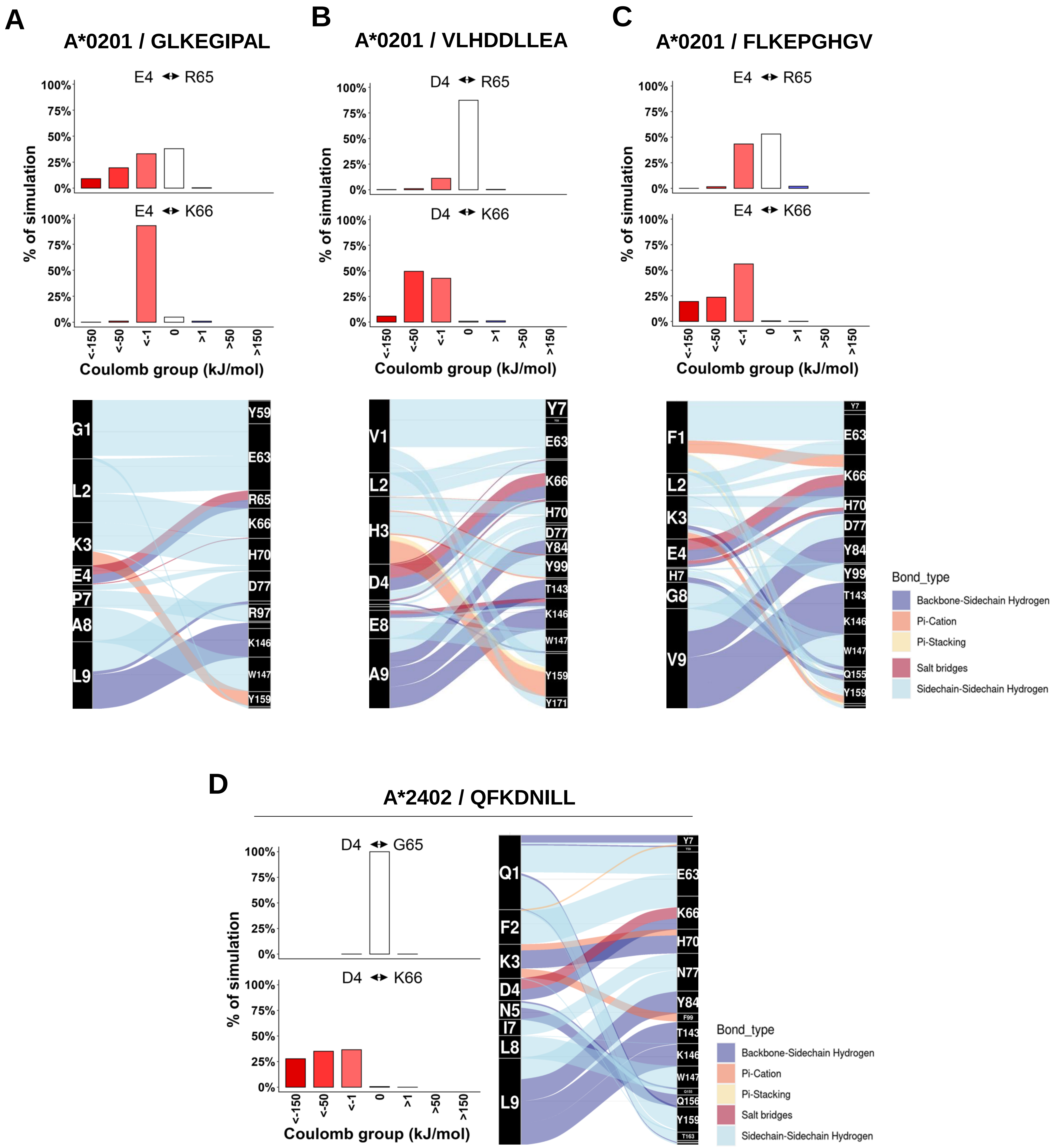

Figure S7. Molecular dynamics simulations show formation of salt bridges with D/E4 residues of peptides bound to A*0201 and A*2402: (A-C) Top panels depict histograms of short-range Coulombic interactions between peptide p4 residues and A*0201 residues R65 or K66 for 3 different A*0201-restricted peptides. Coulombic interactions were computed for each of the conformations extracted from molecular dynamics (MD) simulations and plotted according to the corresponding range of interaction in KJ/mol. Colors indicate the intensity of the interaction, from negative (red) to positive (blue) values. The bottom panels show the corresponding alluvial plots for the same A*0201/peptide complexes. In these plots, the left column indicates the amino acids of the peptide, while the right column indicates amino acids of the HLA-I molecule. The lines connecting the columns indicate interactions between individual pairs of amino acids. The color of the lines indicates the type of interaction as indicated in the legend, and the line width indicates the proportional prevalence over all conformations extracted from the MD simulations. **(D)** Short-range Coulombic interactions between the peptide p4 residue and the A*2402 residues G65 or K66 (left), as well as corresponding alluvial plot (right) for the A*2402-restricted peptide QFKDNVILL.

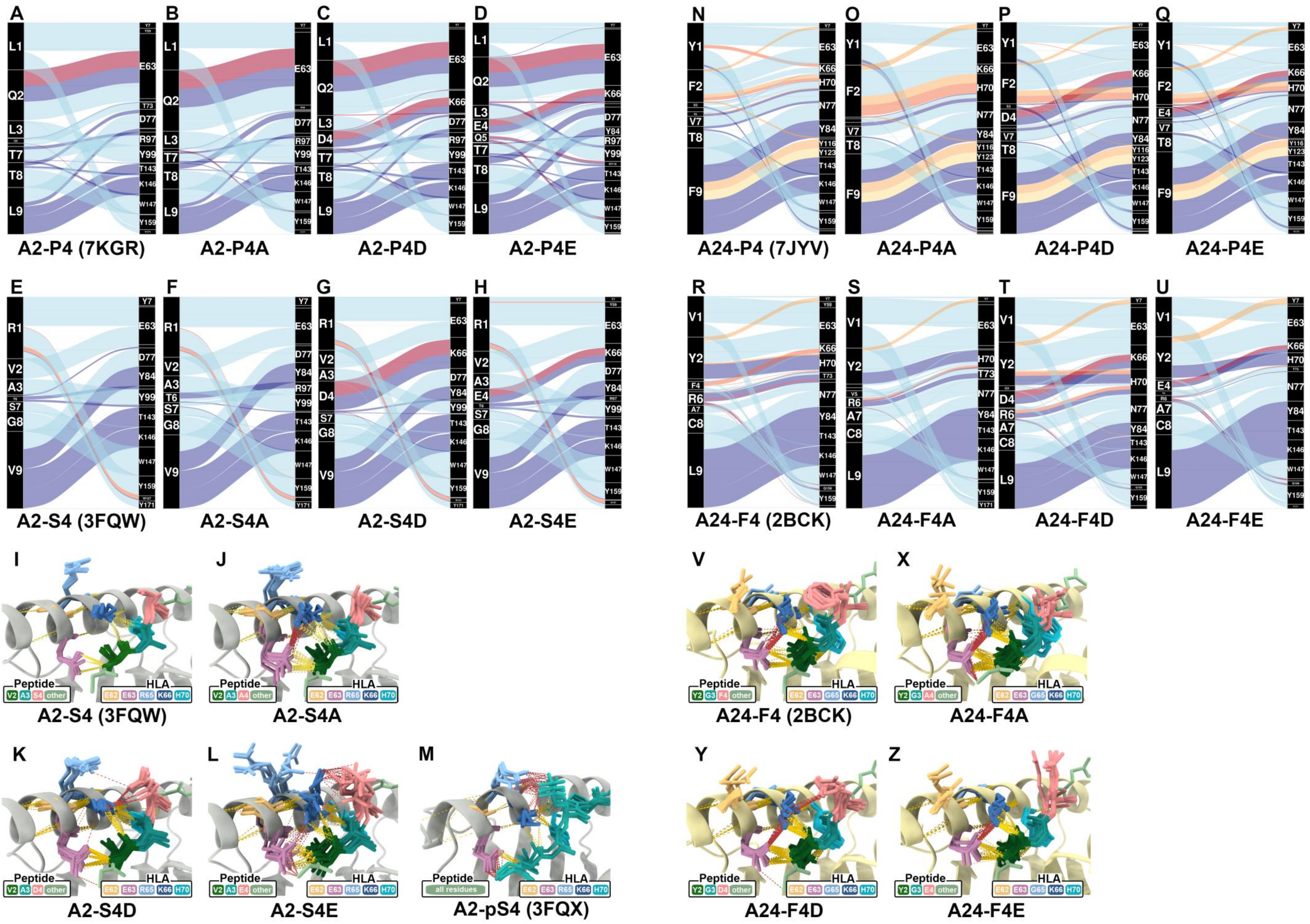

Figure S8. Substitution of other amino acids found at p4 with D/E facilitates the formation of salt bridges with K66: Molecular dynamics simulations (MDs) were performed with 4 crystal structures displaying peptides with other residues at p4 (e.g, P, S or F). For each, mutants replacing the wild-type p4 by A, D or E were also simulated. (A-E) Alluvial plots depicting peptide/HLA interactions (left/right bars, respectively) observed during MDs of complex 7KGR (A*0201/LQLPQGTTL), or mutants. (E-H) Alluvial plots depicting interactions from the MDs of 3FQW (A*0201/RVASPTSGV), or mutants. (I-L) Ensemble of representative conformations from MDs based on 3FQW. (M) Ensemble of conformations from MDs based on 3FQX (A*0201/RVApSPTSGV). (N-Q) Alluvial plots depicting interactions from MDs of complex 7JYV (A*2402/YFSPIRVTF), or mutants. (R-U) Alluvial plots depicting interactions from MDs of 2BCK (A*2402/VYGFVRACL), or mutants. (V-Z) Ensemble of conformations from MDs based on 2BCK.

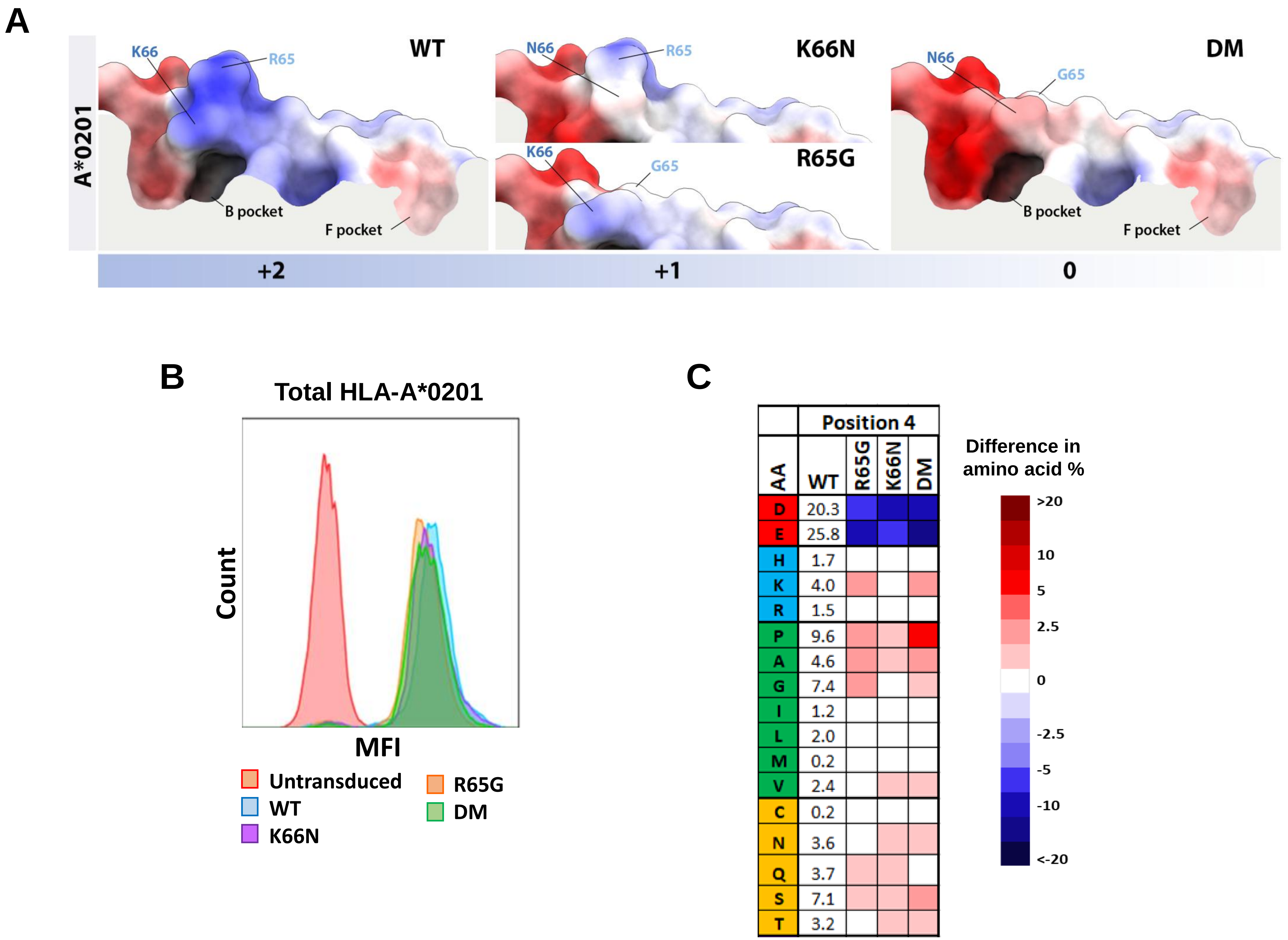

Figure S9. Substitution of R65 and/or K66 in HLA-A*0201 impacts antigen presentation: (A) Comparison of electrostatic potential over the surface of the HLA-I peptide-binding groove between wild-type (WT) A*0201, having two positively charged residues in p66-65, and HLA-A*0201 mutants having only one (K66N, R65G) or no positive charges in this region (DM, double mutant K66N/R65G). **(B)** Flow cytometric analysis showing comparable overall cellular expression of green fluorescent protein (GFP)-linked A2-WT, A2-R65G, A2-K66N and A2-DM molecules in transduced H1975 cells, as determined by median fluorescence intensity (MFI) of GFP, which contrasted with the A*0201 cell surface expression shown in Fig. 2B. **(C)** Heat map depicting the changes in frequencies for all 20 amino acids at position 4 of peptides eluted from A2 mutants compared to wild-type A*02:01.

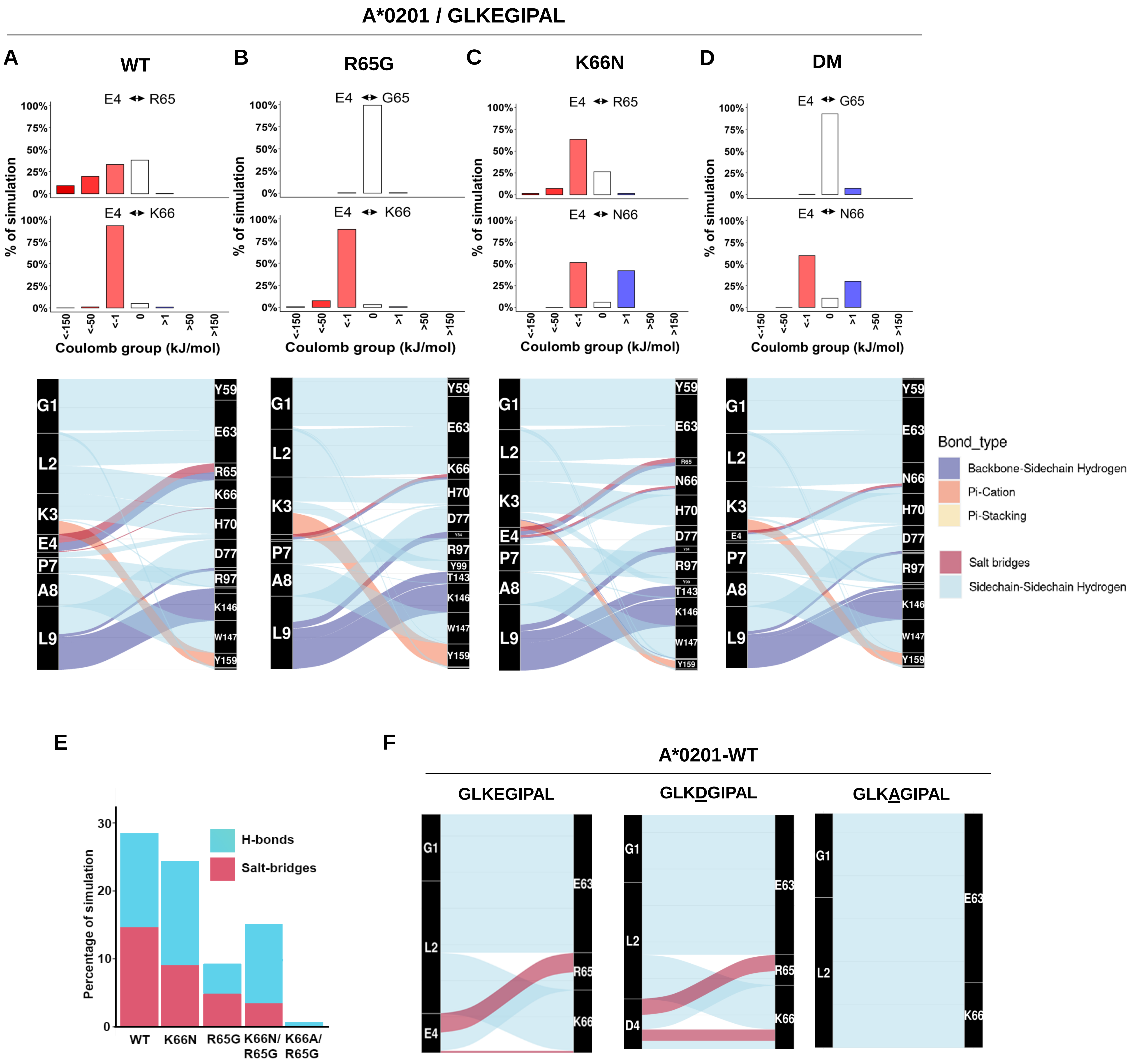

Figure S10. Charged interactions and salt bridges are lost in A*0201 mutants: (A-D) Top panels depict histogram of short-range Coulombic interactions between E4 of the GLKEGIPAL peptide and p65 or p66 of WT HLA-A*0201 (A) or 3 different A*0201 mutants: A2-R65G (B) A2-K66N (C), and A2-DM (R65G/K66N, D). Coulombic interactions were computed with gmx energy (Gromacs Package (*72*)) for each of the conformations extracted from molecular dynamics (MD) simulations and plotted according to the corresponding range of interactions in KJ/mol. Colors indicate the intensity of the interaction, from negative (red) to positive (blue) values. Bottom panels present the corresponding alluvial plots for the same A*0201/peptide complexes. In these plots, the left column indicates the amino acids of the peptide ligand, and the right column indicates amino acids of the A*0201 molecule. The lines connecting the columns indicate interactions between individual pairs of amino acids. The color of the lines represents the type of interaction as indicated in the figure legend, and the line width indicates the proportional prevalence over all conformations extracted from the MD simulations. **(E)** Stacked histogram showing hydrogen bonds (blue) and salt bridges (red) for the GLKEGIPAL peptide bound to WT A*0201 or to each of four A*0201 mutants, as a percentage of all the conformations extracted from each of the MD simulations run. **(F)** Alluvial plots derived from MD simulations with WT A*0201 complexed to GLKEGIPAL, or to the p4-substituted peptides GLKDGIPAL or GLKAGIPAL.

A

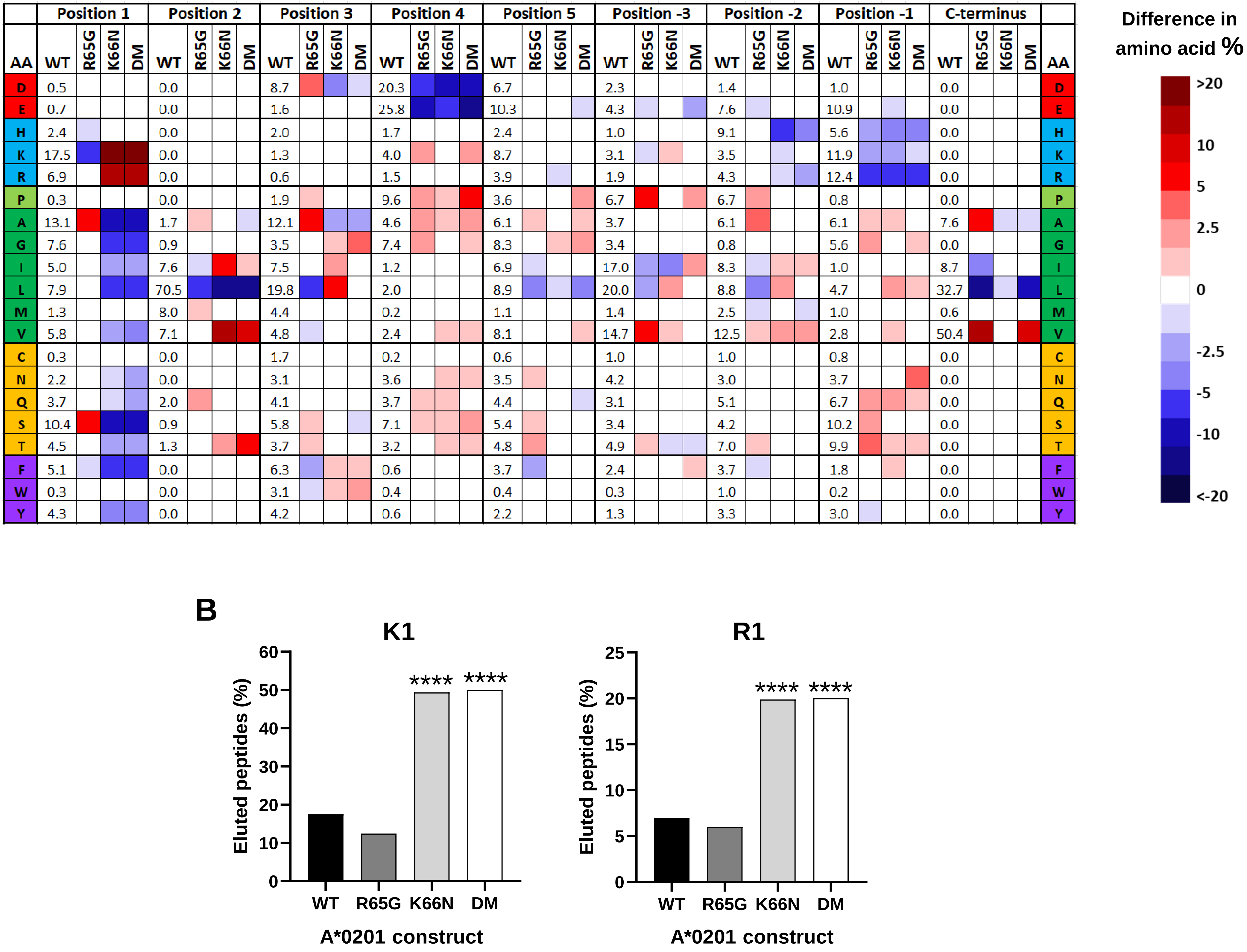

Figure S11. Peptides eluted from mutated A*0201 allotypes demonstrate significantly altered binding motifs: (A) Heat map depicting the changes in frequencies for all 20 amino acids at all positions in peptides eluted from A*0201 mutants R65G, K66N, and DM compared to those eluted from wild-type A*0201 (WT). Amino acid frequencies are shown for peptides eluted from WT A*0201. Increased amino acid frequencies are shown in red and decreased frequencies in blue. Amino acids are grouped according to their biochemical properties: red, negatively charged; blue, positively charged; green, hydrophobic; orange, polar; purple, aromatic. To accommodate peptides of different lengths, positions were counted from the N-terminus (p1 to p5) or from the C-terminus (pΩ to p-3). **(B)** Percentage of K1- or R1-containing peptides eluted from A2-WT, A2-R65G, A2-K66N or A2-DM molecules. **** indicates p≤ 0.0001 using a 2 proportion Z-test.

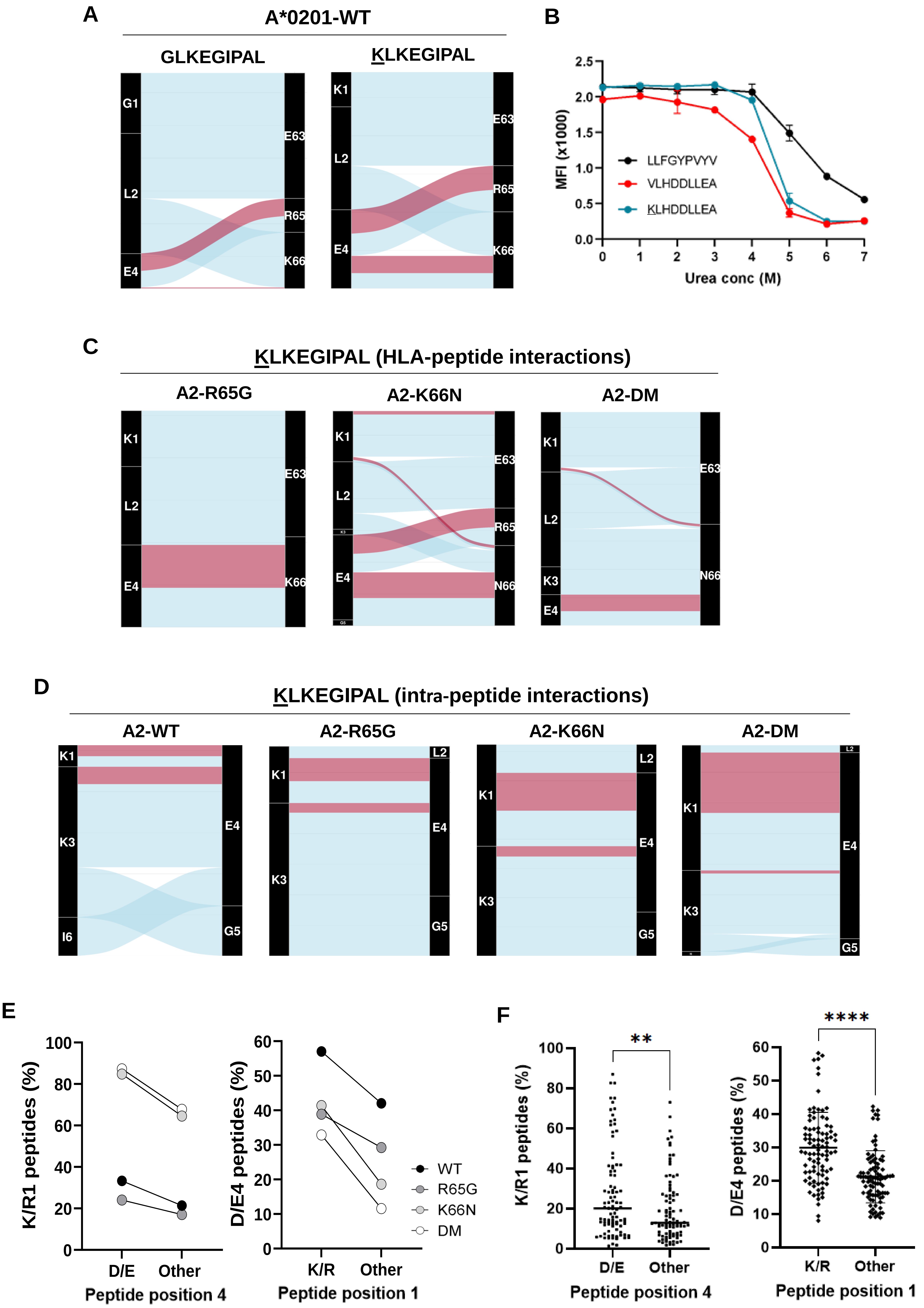

Figure S12. K/R1 peptide residues alter HLA-I/peptide interactions and co-occur with peptide D/E4 residues: (A) Alluvial plots summarizing HLA-I/peptide interactions detected during molecular dynamics (MD) simulations of WT A*0201 bound to GLKEGIPAL or KLKEGIPAL. In these plots, the left column indicates the amino acids of the peptide, and the right column indicates the amino acids of A*0201. The lines connecting the columns indicate interactions between individual pairs of amino acids. The color of the lines indicates the type of interaction (blue, hydrogen bonds; red, salt bridges), and the line width indicates the proportional prevalence over all conformations extracted from the MD simulations. **(B)** Peptide stability assay results of VLHDDLLEA or KLHDDLLEA peptides complexed with WT A*0201, using LLFGYPVYV as a positive control and reference peptide **(C-D)** Alluvial plots as in (A) summarizing (C) HLA-I/peptide interactions or (D) intra-peptide interactions detected during the MD simulations of the KLKEGIPAL peptide bound to WT or mutated A*0201 complexes. **(E)** Graphs depicting the co-occurrence of K/R1 and D/E4 residues in peptides eluted from WT or mutated A*0201 molecules. Left, the proportions of eluted peptides containing D/E4 or other amino acids at position 4 that co-occur with K/R1. Right, the percentages of eluted peptides containing K/R1 or other amino acids at position 1 that co-occur with D/E4. **(F)** Graphs depicting the co-occurrence of K/R1 and D/E4 residues in peptides eluted from 95 different HLA-A, -B, and -C allotypes in the HLAthena database. Left, the proportions of eluted peptides containing D/E4 or other amino acids at position 4 that co-occur with K/R1. Right, the percentages of eluted peptides containing K/R1 or other amino acids at position 1 that co-occur with D/E4. ** indicates p≤ 0.01, **** indicates p≤ 0.0001 using students t-test.

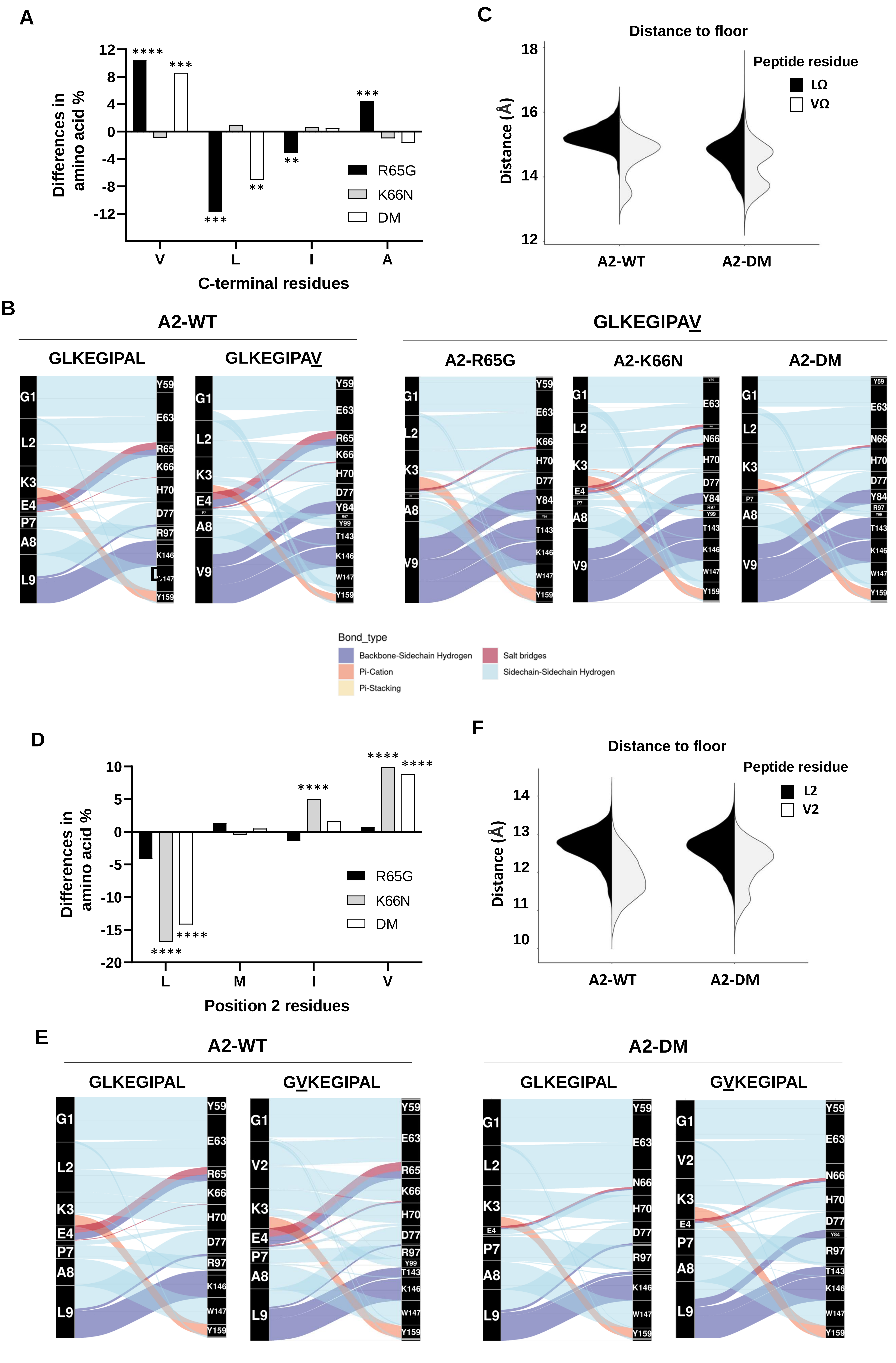

Figure S13. A*0201 mutants show altered position 2 and C-terminal anchor interactions: (A) Differences in the amino acid frequencies of C-terminal (pΩ) anchor residues in peptides eluted from A2-R65G, A2-K66N, or A2-DM mutants compared to A2-WT**. (B)** Alluvial plots summarizing HLA-peptide interactions detected during MD simulations of GLKEGIPAL or GLKEGIPAV peptides bound to WT or mutant A*0201. In these plots, the left column indicates the amino acids of the peptide, and the right column indicates the amino acids of A*0201. The lines connecting the columns indicate interactions between individual pairs of amino acids. The color of the lines indicates the type of interaction as indicated in the legend, and the line width indicates the proportional prevalence over all conformations extracted from the MD simulations. **(C)** Graph showing the distances between the pΩ anchor residue and the WT A*0201 binding cleft floor in MD simulations of GLKEGIPAL or GLKEGIPAV peptides (see Methods). **(D)** Differences in the amino acid frequencies of p2 anchor residues in peptides eluted from A2-R65G, A2-K66N, or A2-DM mutants compared to A2-WT**. (E)** Alluvial plots comparing interactions between GLKEGIPAL and GVKEGIPAL in A2-WT and A2-DM. **(F)** Graph showing the distances between the p2 anchor residue and the WT A*0201 binding cleft floor in MD simulations of GLKEGIPAL or GVKEGIPAL peptides. ** indicates p≤ 0.01, *** indicates p ≤ 0.001, **** indicates p ≤ 0.0001 using a 2 proportion Z-test comparing amino acid frequencies of peptides eluted from mutant A2 molecules compared to A2-WT molecules.

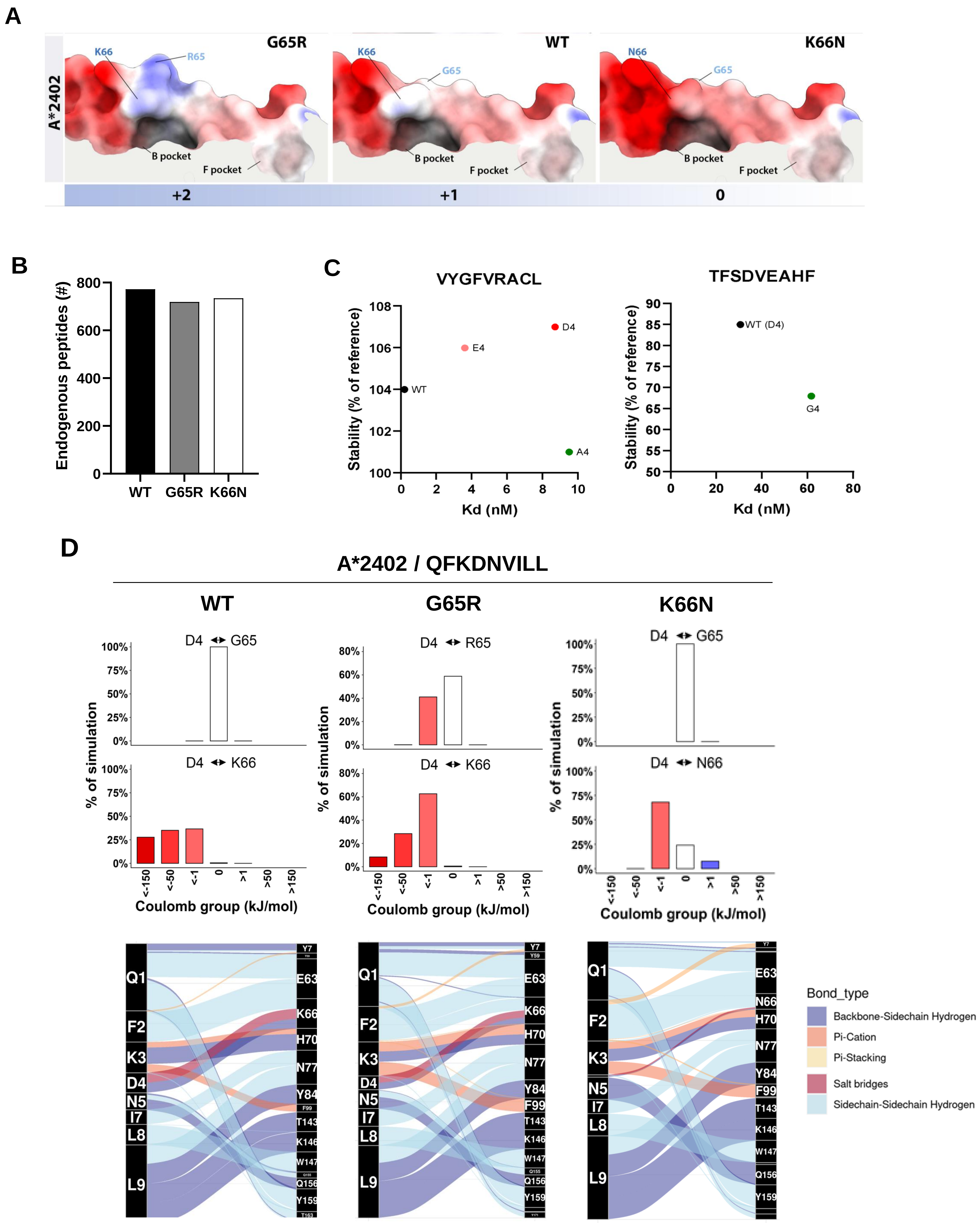

Figure S14. Comparison of HLA-I/peptide interactions in WT and mutant A*2402 molecules: (A) Comparison of electrostatic potential over the HLA-I surface between WT A*2402, containing one positively charged residue in p65/66, and two A*2402 mutants having two positively charged residues (A24-G65R) or no positive charges (A24-K66N) in this region. **(B)** Comparable numbers of endogenous peptides (bound to endogenous allotypes HLA-A*0101, HLA-B*4101, and HLA-C*1701 allotypes) were eluted from H1975 cells transduced to express A24-WT, A24-G65R and A24-K66N molecules. **(C)** Peptide binding and stability assay results performed on WT A*2402 using 2 different A*2402-restricted peptide backbones with the indicated p4 residue substitutions. Relative stability is indicated on the y-axis and measured peptide binding affinity is shown on the x-axis. Stability values were calculated compared to the reference peptide TYTQDFNKF. **(D)** Top panels depict histograms of short-range Coulombic interactions between the QFKDNVILL peptide D4 residue and the A*2402 residues p65 or p66, for A24-WT, A24-G65R and A24-K66N molecules. Coulombic interactions were computed for each of the conformations extracted from the MD simulations and plotted according to the corresponding range of interaction in KJ/mol. Colors indicate the intensity of the interaction, from negative (red) to positive (blue) values. Bottom panels show the corresponding alluvial plots for the same complexes. In these plots, the left column indicates the amino acids of the peptide, and the right column indicates the amino acids of A*2402. The lines connecting the columns indicate interactions between individual pairs of amino acids. The color of the lines indicates the type of interaction as indicated in the legend, and the line width indicates the proportional prevalence over all conformations extracted from the MD simulations.

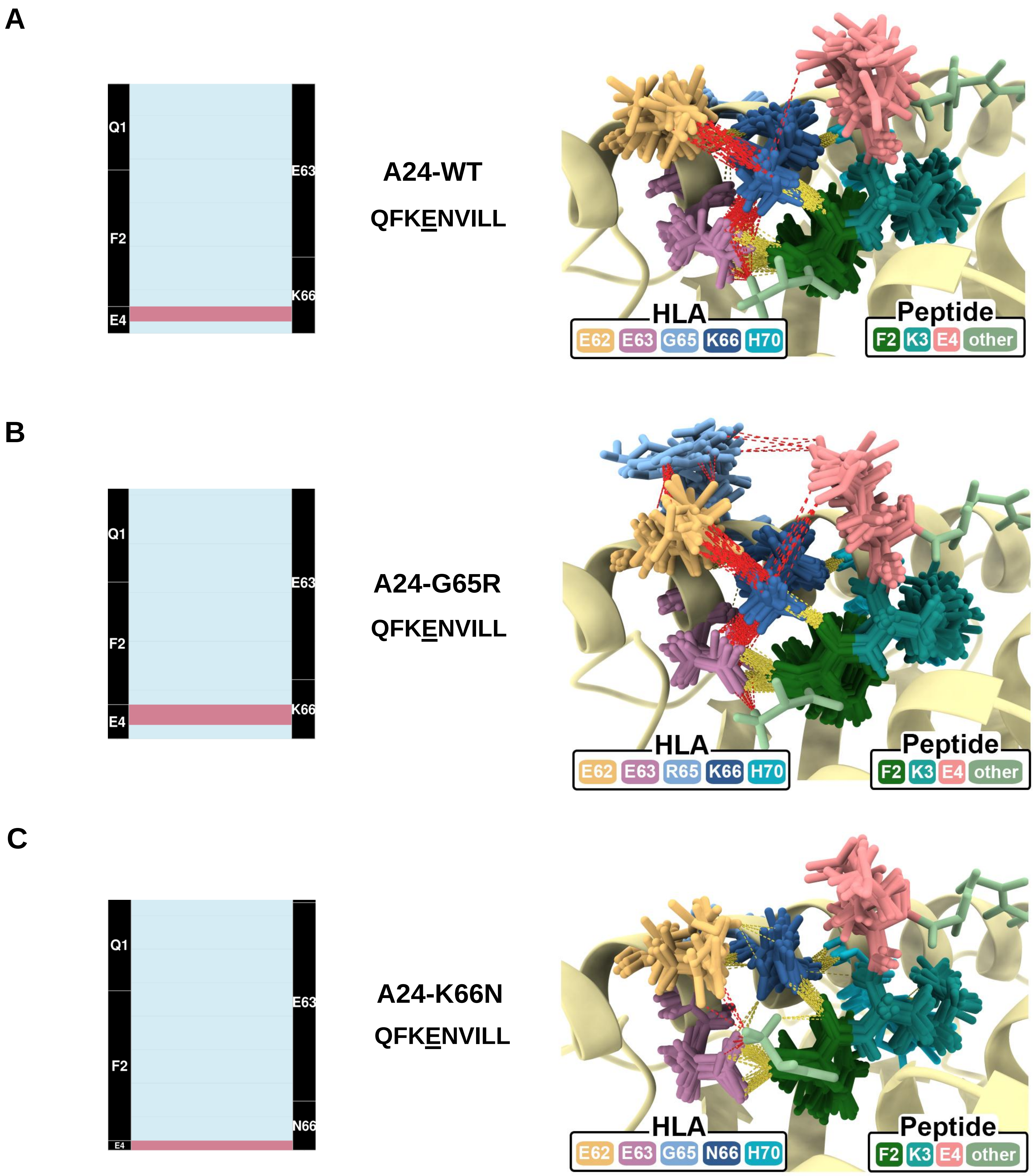

Figure S15. Comparison of interactions of WT and mutant A*2402 allotypes complexed to QFKENVILL: **(A-C)** Left side, Alluvial plots generated from MD simulations of the p4-substituted peptide QFKENVILL bound to WT or mutated A*2402. In these plots, the left column indicates the amino acids of the peptide, and the right column indicates the amino acids of A*2402. The lines connecting the columns indicate interactions between individual pairs of amino acids. The color of the lines indicates the type of interaction (blue, hydrogen bonds; red, salt bridges), and the line width indicates the proportional prevalence over all conformations extracted from the MD simulations. Right side, Ensemble of representative conformations extracted from multiple MD simulations of the QFKENVILL peptide bound to A24-WT, A24-G65R and A24-K66N. Alternative conformations of the side chains of peptide residues 2, 3 and 4, as well as A*2402 residues 62, 63, 65, 66 and 70 are displayed using sticks, and colored by amino acid position according to the legend. For other peptide positions, only main chain atoms are depicted. For other A*2402 residues, only the main chain of a single conformation is depicted. In all panels, yellow dashed lines indicate hydrogen bonds and red dashed lines indicate salt bridges.

A

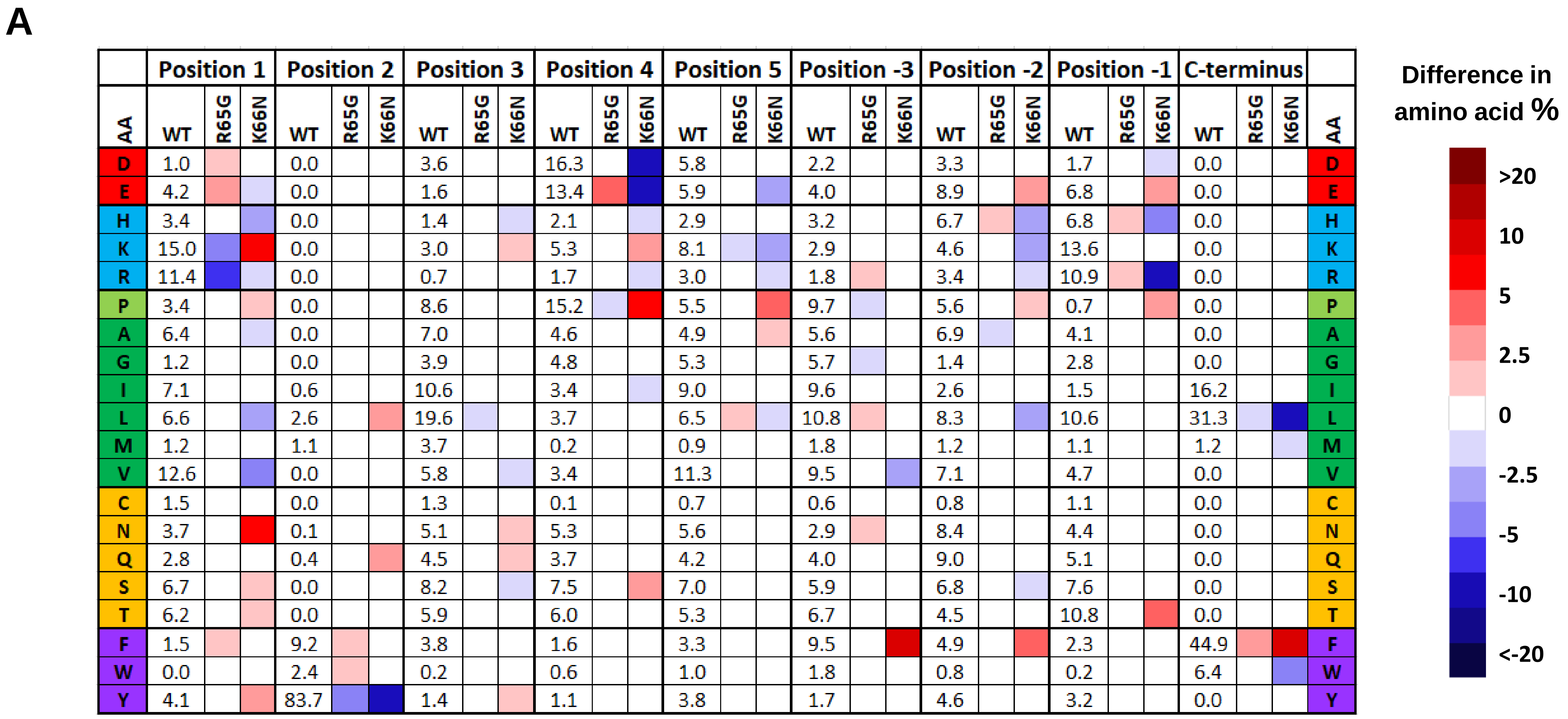

	Position 1			Position 2			Position 3			Position 4			Position 5			Position -3			Position -2			Position -1			C-terminus			
AA	WT	R65G	K66N	WT	R65G	K66N	WT	R65G	K66N	WT	R65G	K66N	WT	R65G	K66N	WT	R65G	K66N	WT	R65G	K66N	WT	R65G	K66N	WT	R65G	K66N	AA
D	1.0			0.0			3.6			16.3			5.8			2.2			3.3			1.7			0.0			D
E	4.2			0.0			1.6			13.4			5.9			4.0			8.9			6.8			0.0			E
H	3.4			0.0			1.4			2.1			2.9			3.2			6.7			6.8			0.0			H
K	15.0			0.0			3.0			5.3			8.1			2.9			4.6			13.6			0.0			K
R	11.4			0.0			0.7			1.7			3.0			1.8			3.4			10.9			0.0			R
P	3.4			0.0			8.6			15.2			5.5			9.7			5.6			0.7			0.0			P
A	6.4			0.0			7.0			4.6			4.9			5.6			6.9			4.1			0.0			A
G	1.2			0.0			3.9			4.8			5.3			5.7			1.4			2.8			0.0			G
I	7.1			0.6			10.6			3.4			9.0			9.6			2.6			1.5			16.2			I
L	6.6			2.6			19.6			3.7			6.5			10.8			8.3			10.6			31.3			L
M	1.2			1.1			3.7			0.2			0.9			1.8			1.2			1.1			1.2			M
V	12.6			0.0			5.8			3.4			11.3			9.5			7.1			4.7			0.0			V
C	1.5			0.0			1.3			0.1			0.7			0.6			0.8			1.1			0.0			C
N	3.7			0.1			5.1			5.3			5.6			2.9			8.4			4.4			0.0			N
Q	2.8			0.4			4.5			3.7			4.2			4.0			9.0			5.1			0.0			Q
S	6.7			0.0			8.2			7.5			7.0			5.9			6.8			7.6			0.0			S
T	6.2			0.0			5.9			6.0			5.3			6.7			4.5			10.8			0.0			T
F	1.5			9.2			3.8			1.6			3.3			9.5			4.9			2.3			44.9			F
W	0.0			2.4			0.2			0.6			1.0			1.8			0.8			0.2			6.4			W
Y	4.1			83.7			1.4			1.1			3.8			1.7			4.6			3.2			0.0			Y

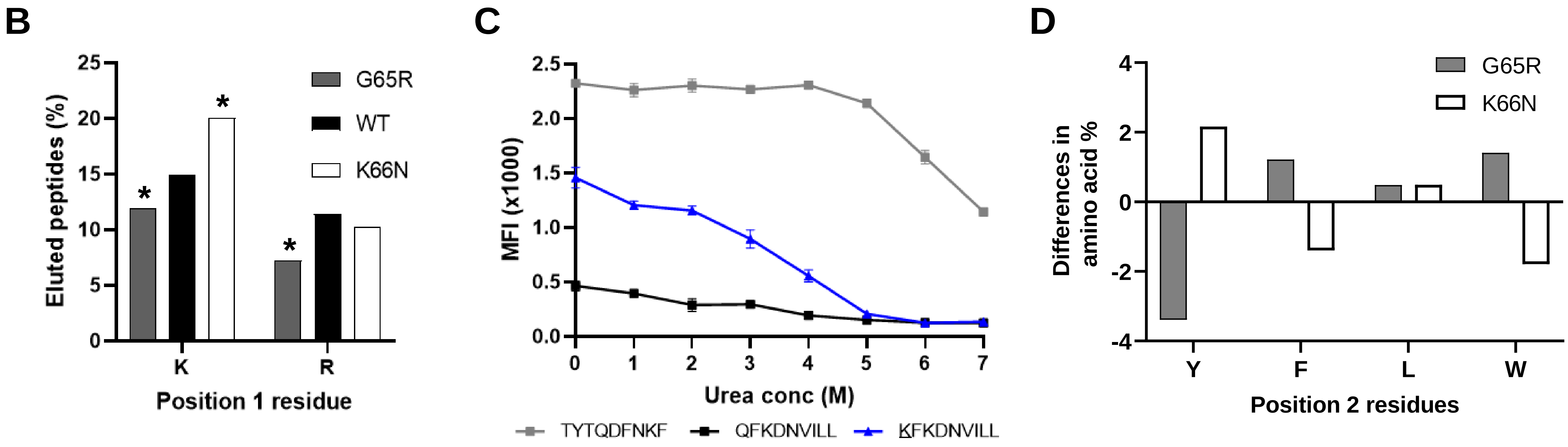

Figure S16. Peptides eluted from mutated A*2402 allotypes demonstrate significantly altered binding motifs: (A) Heat map depicting the changes in frequencies for all 20 amino acids at all peptide positions for peptides eluted from A*2402 mutants compared to wild-type A*2402. Increased amino acid frequencies are shown in red and decreased frequencies in blue. Amino acids are grouped according to their biochemical properties: red, negatively charged; blue, positively charged; green, hydrophobic; orange, polar; purple, aromatic. To accommodate peptides of different lengths, positions were counted from the N-terminus (p1 to p5) or from the C-terminus (pΩ to p-3). **(B)** Proportion of peptides eluted from A24-WT, A24-G65R, and A24-K66N molecules that contain K or R at peptide position 1. * indicates p≤ 0.05 using a 2 proportion Z-test. **(C)** Peptide binding stability assays comparing QFKDNVILL and KFKDNVILL peptides complexed to WT A*2402, using TYTQDFNKF as a positive control and reference peptide. **(D)** Differences in the amino acid frequencies of p2 anchor residues in peptides eluted from A24-G65R and A24-K66N, compared to peptides eluted from A24-WT.

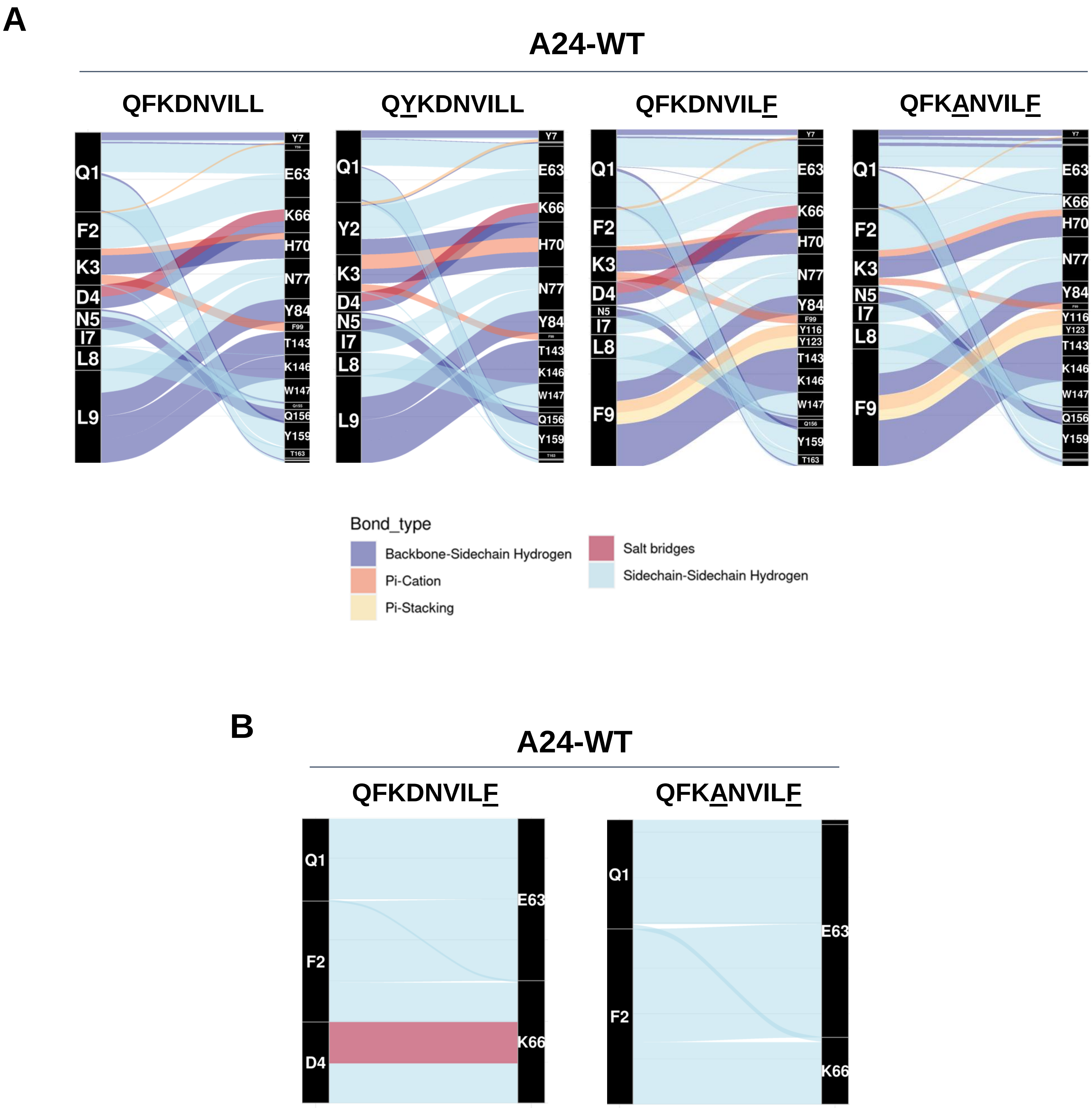

Figure S17. Y2 and FΩ form stronger interactions with HLA-A*2402 compared with other primary anchors: (A) Alluvial plots of molecular interactions between peptide and HLA residues in the binding groove of A*2402 WT bound to QFKDNVILL or residue-substituted peptides QYKDNVILL, QFKDNVILF and QFKANVILF. In these plots, the left column indicates the amino acids of the peptide, while the right column indicates amino acids of the HLA-I molecule. The lines connecting the columns indicate interactions between individual pairs of amino acids. The color of the lines indicates the type of interaction as indicated in the legend, and the line width indicates the proportional prevalence over all conformations extracted from the MD simulations. **(B)** Simplified alluvial plots derived from MD simulations with WT A*2402 complexed to QFKDNVILF compared to the p4-substituted peptide QFKANVILF. The color of the lines indicates the type of interaction (blue, hydrogen bonds; red, salt bridges), and the width indicates the proportional prevalence over all the conformations extracted from the MD simulations.

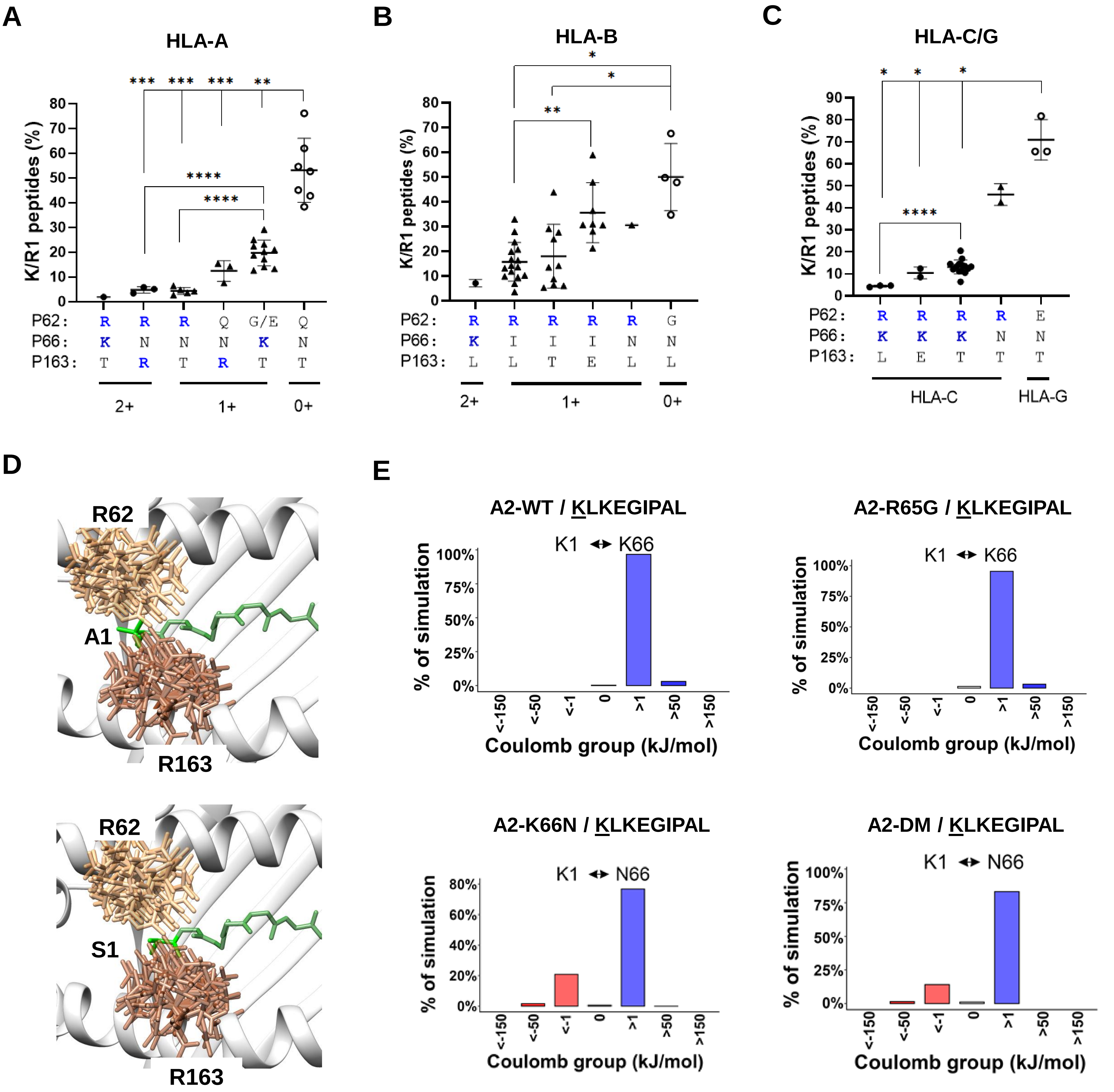

Figure S18. The presence of positively charged amino acids at peptide position 1 strongly correlates with their absence at HLA-I positions 62, 66 and 163: (A-C) The proportion of K/R1 peptide ligands eluted from individual (A) HLA-A, (B) HLA-B, or (C) HLA-C and HLA-G allotypes, grouped by their configuration of amino acids in positions 62, 66 and 163. Symbols indicate allotypes with no positively charged residues at p62, p66 or p163 (open circles), allotypes containing one positively charged residue at these positions (filled triangles), and allotypes containing two positively changed residues at these positions (filled circles). **(D)** Computational modeling shows lack of overlap of conformational possibilities between p1 peptide residues with small polar side chains alanine A1 (top) or serine S1 (bottom) in light green, and HLA-I residues R62 (light tan) and R163 (dark tan). **(E)** Coulombic interactions between the peptide K1 residue and the A*0201 p66 residue, derived from MD simulations of the KLKEGIPAL peptide bound to A*0201 WT (top left panel), A2-R65G (top right panel), A2-K66N (bottom left panel) and A2-DM (bottom right panel). Coulombic interactions were computed for each of the conformations extracted from the MD simulations and plotted according to the corresponding range of interaction in KJ/mol. Colors indicate the intensity of the interaction, from negative (red) to positive (blue) values. For (A-C), Brown-Forsythe ANOVA tests showed significant difference between means (p≤0.0001), and multiple comparisons using Dunnett's T3 test were also performed to assess the significance of differences between specific groups. * indicates p≤ 0.05, ** indicates p≤ 0.01, *** indicates p≤ 0.001, **** indicates p≤ 0.0001.

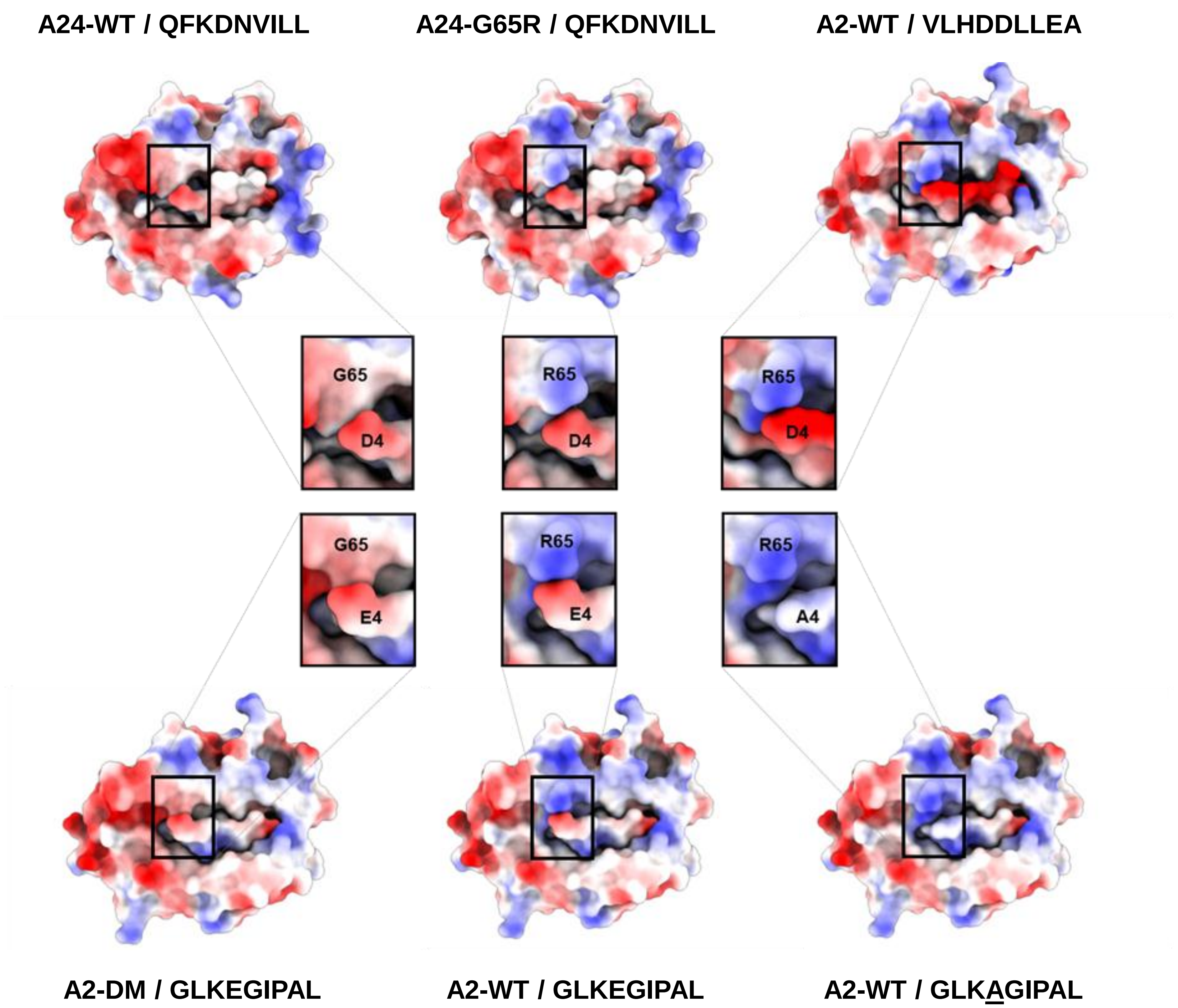

Figure S19. Charged HLA-I/peptide surface interactions involving peptide position 4 are visible to T-cell receptors: Computational modeling showing a comparison of electrostatic potential over the TCR recognition surface of six different HLA-I/peptide complexes: A24-WT and A24-G65R molecules bound to QFKDNVILL; A2-WT bound to VLHDDLLEA; A2-WT and A2-DM bound to GLKEGIPAL; and A2-WT bound to the p4 residue-substituted peptide GLKAGIPAL. Close-up inserts for each complex highlight the surface charges contributed by D, E, or A residues at peptide position 4, and G or R residues at HLA-I position 65. Red color indicates regions with a net negative charge and blue color indicates a net positive charge.

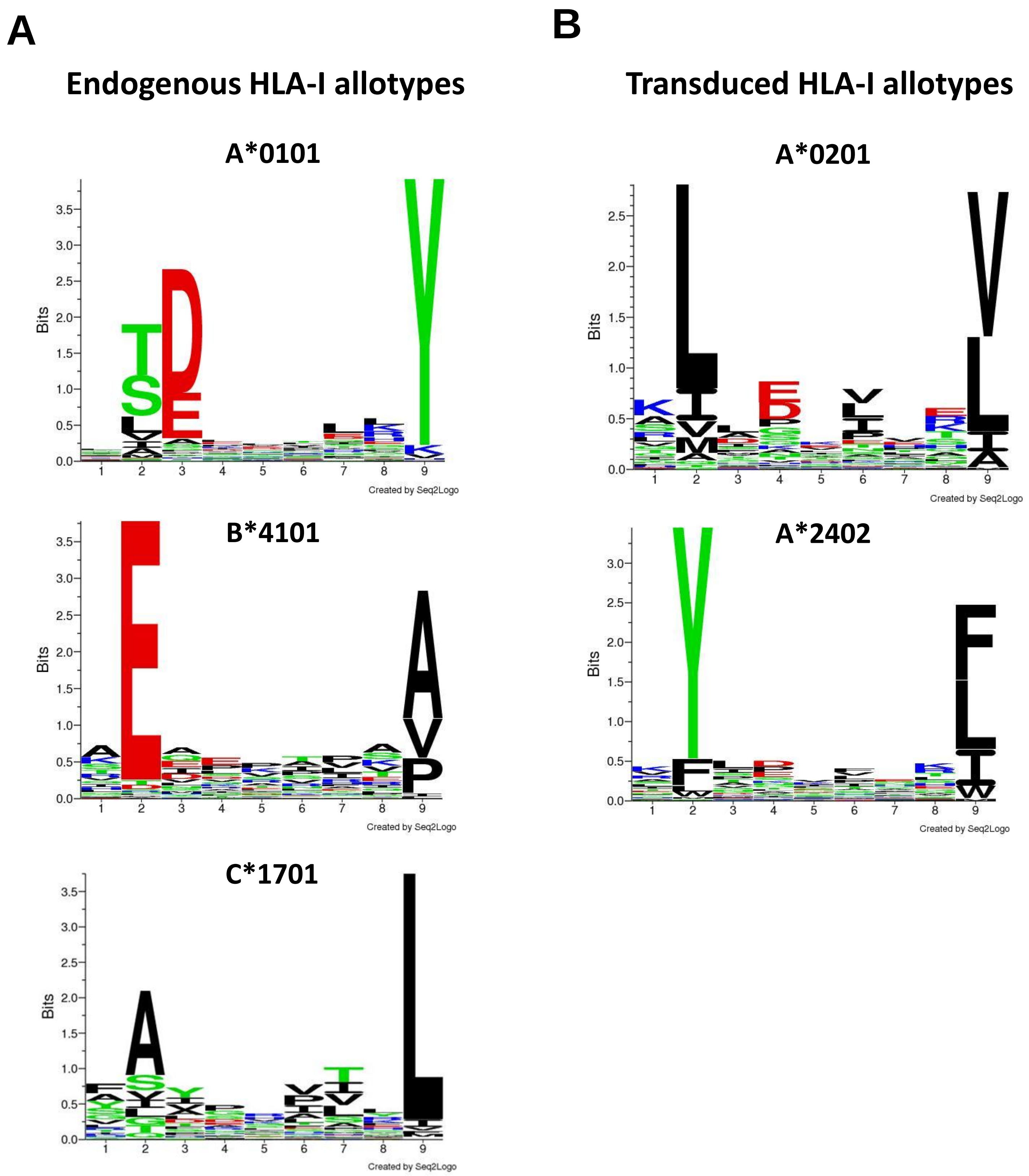

Figure S20. Comparison of peptide binding motifs for endogenous H1975 HLA-I allotypes and transduced HLA-I molecules: (A) Peptide binding motifs for HLA-I allotypes expressed endogenously by the H1975 lung cancer cells line: A*0101, B*4101 and C*1702. The A*0101 and B*4101 peptide motifs were derived from H1975-eluted peptides, and the C*1701 motif was derived from the HLAthena peptide ligand dataset. **(B)** Peptide binding motifs for the transduced wild-type HLA-I allotypes A*0201 and A*2402, which are easily distinguishable from those of the endogenous HLA-I allotypes.

Country/Region	Ethnicity (USA)	Phenotype frequency	Allele frequency	Sample size
Russia/Karelia		50.5	0.2963	1 075
USA	Caucasian	47.6	0.2755	1 242 890
Ireland/Northern		47.3	0.274	1 000
Germany		46.2	0.2667	39 689
Spain		41.8	0.2368	4 335
USA	Hispanic	37.5	0.2095	146 714
China/Hong Kong		34.3	0.1891	7 595
Columbia		29.6	0.1613	1 463
South Korea		29.1	0.158	4 128
China/Jiangsu Han		24.3	0.13	3 238
USA	African-American	23.1	0.1235	416 581
Japan		21.8	0.1162	18 604

Table S1. **Worldwide phenotypic and allele frequencies of HLA-A*0201**: Phenotype and allele frequencies for HLA-A*0201 listed in order of prevalence in diverse ethnic populations from the United States and from several other countries worldwide, including China, Japan, Germany and South Korea.

Country/Region	Ethnicity (USA)	Phenotype frequency	Allele frequency	Sample size
Japan		59.7	0.3648	18 604
USA	Filipino	42.3	0.2399	50 614
USA	Pacific Islander	42.1	0.2393	11 499
South Korea		39.3	0.2209	4 128
Malaysia		35.7	0.1977	951
India/Tamil Nadu		30	0.1629	2 492
China/Hubei Han		29.2	0.159	3 732
China/Hong Kong		27.3	0.1472	7 595
USA	Hispanic	24.6	0.1316	146 714
USA	Middle Eastern	22.1	0.1174	70 890
Germany		18.1	0.0954	39 689
USA	Caucasian	16.3	0.0846	1 242 890

Table S2. **Worldwide phenotypic and allele frequencies of HLA-A*2402**: Phenotype and allele frequencies for HLA- A*2402 listed in order of prevalence in diverse ethnic populations from the United States and from several other countries worldwide, including China, Japan, Germany and South Korea.

Species	Allele	Sequence
		60 65 70 75
Canine	DLA-88*001:01	EYWDRET**R**TAKETAQRYR
	DLA-88*005:01	EYWDRQT**R**NFKETARNFR
	DLA-88*34:01	EYWDGET**RK**VKETAQLYR
	DLA-88*046:01	EYWDGET**RK**VKETAQVYR
	DLA-88*501:01	EYWDPQT**R**TIKETARTFR
	DLA-88*508:02	EYWDGET**RK**VKETAQVYR
Murine	H-2-Kb	EYWERETQ**K**AKGNEQSFR
	H-2-Db	EYWERETQ**K**AKGGEQWFR
	H-2-Kd	EYWEEQTQ**R**AKSDEQWFR
	H-2-Kk	EYWERNTQIAKGNEQIFR
	H-2-Ld	EYWERITQIAKQQEQWFR
	H-2-Dd	EYWERET**RR**AKGNEQSFR
Bovine	BoLA-1*019:01	EYWDRET**R**ISKENTLVYR
	BoLA-2*012:01	EYWDRET**R**NFKDTAQTFR
	BoLA-2*016:01	EYWDRNT**R**IYKDTAQTFR
	BoLA-2*018:01	EYWDRNT**R**IYKDTAQIFR
	BoLA-3*002:01	EYWDQET**R**KAKGNAQFFR
	BoLA-3*017:01	EYWDRETQ**R**AKGNAQIFR
	BoLA-4*024:01	EYWDEQT**R**IVKDTAQTFR
	BoLA-6*013:01	EYWDRETQISKENALWYR
	BoLA-6*014:02	EYWDRETQISKENALKYR
Rhesus monkey	Mamu-A1*001:01	EYWDRET**R**NMKTETQNAP
	Mamu-A1*002:01	EYWDRET**R**NMKAETQNAP
	Mamu-A1*011:01	EYWDRET**R**ISKANTQTYR
	Mamu-B*001:01	EYWDRET**RK**AKGNAQTDR
	Mamu-B*003:01	EYWEEET**R**NAKGHAQTDR
	Mamu-B*017:01	EYWEEAT**RR**AKEAAQTHR
	Mamu-B*039:01	EYWEEET**RR**AKETAQTFR
	Mamu-B*083:01	EYWEEQT**R**NSKARAQTDR

Table S3. Animal MHC class I molecules conserve positively charged residues at p65 and p66: Sequence alignment of positions 58-77 of a selection of MHC class I molecules from dog, mouse, cattle and rhesus monkey show conservation of arginine (R) and lysine (K) at positions 65 and/or 66 (indicated in blue font).

Supplemental Methods

Molecular dynamics (MD) simulations

Input structures for MD simulations were pre-processed with the PDB2PQR webserver [1]. Structures were fixed as needed, protonated at pH 7.0 using the PROPKA algorithm [2], and atom distances and hydrogen bonding networks were optimized according to the CHARMM force field. All MD simulations were performed with the GROMACS 2021 package [3] using the CHARMM36 force field [4] and the TIP3 water model. A cubic box was defined with at least 15 Å of liquid layer around the structure, with periodic boundary conditions. Sodium (Na+) and chloride (Cl−) counter-ions were added to neutralize the system, with a final concentration of 0.15 mol/L. The algorithms v-rescale (with tau-t = 0.1 ps) and Parrinello-Rhaman (with tau-p = 2 ps) were used for temperature and pressure coupling, respectively. A cutoff value of 1.2 nm was used for both the van der Waals and Coulomb interactions, with Fast Particle Mesh Ewald electrostatics (PME).

The production stage of each MD simulation was preceded by (i) three steps of Energy Minimization (EM) and (ii) eight steps of Equilibration (EQ), as previously described [5-6]. Briefly, the first EM step is conducted using the steepest-descent algorithm and position restraints on all amino acid's heavy atoms (5000 kJ−1mol−1nm−1), allowing relaxation of the solvent only. The second EM step involves the same algorithm, but no restraint. The third EM step uses the conjugate-gradient algorithm, without restraint, to further relax the protein. The EQ phase starts at a temperature of 310 K, which is maintained for 300 ps, applying position restraints on the protein heavy atoms (5000 kJ−1mol−1nm−1). This step allows solvation layers to form without affecting the HLA-I folding. Temperature is then reduced to 280 K, and position restraints are gradually reduced. This process is followed by a progressive temperature increase, up to 300 K. Together, these EQ steps constitute the first 500 ps of each MD simulation. During the production stage, the temperature remains constant at 300 K. To improve performance, the HLA receptor was truncated at residue 180, as previously described in Abella et al., 2020 [7]. This resulted in a system composed only of the HLA-I binding site (domains alpha-1 and alpha-2) and the bound peptide. To ensure maintenance of the binding cleft overall conformation, position restraints were applied to the alpha carbons of residues in the beta-sheet floor [7].

The main MD protocol consisted of running five independent simulations of 20 ns for each HLA-I/peptide complex of interest. The total sampling obtained with this protocol is equivalent to one MD of 100 ns per complex, but produces better thermodynamical descriptors by having replicated simulations [8-10]. To confirm the reliability of the main protocol, some complexes were also simulated with alternative protocols. These test protocols included a single longer simulation (e.g., 200 ns), as well as short simulations with higher number of replicates (e.g., 10 simulations of 20 ns). Finally, test simulations with an alternative force field (e.g., Gromos 54a7) and water model (e.g., SPC) were executed. Despite small fluctuations in the frequencies of observed interactions, the main observations were consistent across protocols.

Molecular dynamics (MD) analyses

Raw trajectory files were post-processed with gromacs tools such as trjconv to perform rotational and translational alignment of sampled conformations, correct effects of periodic boundary conditions, and remove water molecules. The python package MDtraj [11] was used to

read and aggregate conformations sampled across replicates, recovering a total of 49,750 conformations per complex. These conformations were used as input to compute several types of non-covalent interactions between the peptide and the HLA-I molecule. The python package GetContacts (https://getcontacts.github.io/) and multiple gromacs tools (e.g., energy, rmsf, rmsd) were used in these analyses. Resulting data was summarized in plots obtained with custom scripts, relying on standard packages for python (e.g., numpy, pandas, matplotlib, seaborn) and R (e.g., ggplot2, RcolorBrewer and ggalluvial).

To create an ensemble of representative conformations for visual inspection we used a data-driven protocol previously implemented by our team [12] with algorithms from the scikit-learn package. Briefly, we computed the minimal distances between all possible pairs of amino acids between peptide and HLA-I amino acid residues, for all HLA-I/peptide conformations extracted from the MD simulations of a given complex. The distance between the closest pair of heavy-atoms belonging to each pair of amino acids was used as features for a Principal Component Analysis (PCA). This dimensionality reduction step was followed by clustering of states, using the Elbow method of scikit-learn to determine the ideal number of clusters (K). Finally, representative members of each cluster were identified with K-means and extracted to build the final ensembles for visual inspection. Images of ensembles were produced with ChimeraX. Representations of hydrogen bonds and salt bridges were added to the structures using the H-bond tool under structure analyses. For hydrogen bonds, a distance tolerance of 0.4 Å and angle tolerance of 20° were used. A more permissive distance tolerance of 1.8 Å was used for salt bridges. Note that these calculations are performed over a small sample of representative conformations, for visual inspection purposes, and do not reflect the full range of interactions over the simulated period. A more complete description of the occurrence and prevalence of these interactions is provided by the alluvial plots shown.

Minimal distances between residues of interest were computed with MDtraj, as the distance between the closest pair of heavy atoms belonging to an amino acid of the peptide and an amino acid of the HLA-I molecule, and plotted using seaborn.violinplot. This approach was used to analyze the distance between peptide p4 and key HLA-I residues (for example, as in Figure 1G), as well as to analyze the distance from the center of the HLA β-sheet floor (p98) to peptide pΩ or p2.

Supplemental References

1. TJ Dolinsky, JE Nielsen, JA McCammon, NA Baker, PDB2PQR: an automated pipeline for the setup of Poisson-Boltzmann electrostatics calculations. Nucleic Acids Res 32, W665–667 (2004).

2. MH Olsson, CR Søndergaard, M Rostkowski, JH Jensen, PROPKA3: Consistent Treatment of Internal and Surface Residues in Empirical pKa Predictions. J Chem Theory Comput. 7, 525–537 (2011).

3. S Pronk, et al., GROMACS 4.5: a high-throughput and highly parallel open source molecular simulation toolkit. Bioinformatics 29, 845–854 (2013).

4. RB Best, et al., Optimization of the additive CHARMM all-atom protein force field targeting improved sampling of the backbone , and side-chain (1) and (2) dihedral angles. J Chem Theory Comput. 8, 3257–3273 (2012).

5. T Arns, et al., Structural Modeling and Molecular Dynamics of the Immune Checkpoint Molecule HLA-G. Front Immunol 11, 575076 (2020).

6. D Devaurs, et al., Coarse-Grained Conformational Sampling of Protein Structure Improves the Fit to Experimental Hydrogen-Exchange Data. Front Mol Biosci 4, 13 (2017).

7. JR Abella, et al., Markov state modeling reveals alternative unbinding pathways for peptide MHC complexes. Proc Natl Acad Sci U S A 117, 30610–30618 (2020).

8. S Wan, B Knapp, DW Wright, CM Deane, PV Coveney, Rapid, Precise, and Reproducible Prediction of Peptide-MHC Binding Affinities from Molecular Dynamics That Correlate Well with Experiment. J Chem Theory Comput. 11, 3346–3356 (2015).

9. AP Bhati, S Wan, Y Hu, B Sherborne, PV Coveney, Uncertainty Quantification in Alchemical Free Energy Methods. J Chem Theory Comput. 14, 2867–2880 (2018). 918

10. N Plattner, F Noé, Protein conformational plasticity and complex ligand-binding kinetics explored by atomistic simulations and Markov models. Nat Commun 6, 7653 (2015).

11. RT McGibbon, et al., MDTraj: A Modern Open Library for the Analysis of Molecular Dynamics Trajectories. Biophys J 109, 1528–1532 (2015).

12. S Hall-Swan, et al., DINC-COVID: A webserver for ensemble docking with flexible SARS-CoV-2 proteins. bioRxiv (2021).

Supplemental Video Captions

Suppl Video 1: Video from MD simulation of GLKEGIPAL bound to A2-WT.

Suppl Video 2: Video from MD simulation of QFKDNVILL bound to A24-WT.

Suppl Video 3: Video from MD simulation of GLKEGIPAL bound to A2-R65G.

Suppl Video 4: Video from MD simulation of GLKEGIPAL bound to A2-K66N.

Suppl Video 5: Video from MD simulation of GLKEGIPAL bound to A2-DM.

Suppl Video 6: Video from MD simulation of GLKDGIPAL bound to A2-WT.

Suppl Video 7: Video from MD simulation of GLKAGIPAL bound to A2-WT.

Suppl Video 8: Video from MD simulation of KLKEGIPAL bound to A2-WT.

Suppl Video 9: Video from MD simulation of KLKEGIPAL bound to A2-R65G.

Suppl Video 10: Video from MD simulation of KLKEGIPAL bound to A2-K66N.

Suppl Video 11: Video from MD simulation of KLKEGIPAL bound to A2-DM.

Suppl Video 12: Video from MD simulation of QFKDNVILL bound to A24-G65R.

Suppl Video 13: Video from MD simulation of QFKDNVILL bound to A24-K66N.
